# Supplementary material for: A biocompatible electrolyte enables highly reversible Zn anode for zinc ion battery
Source: Nat Commun. 2023 Oct 16;14:6526. doi: 10.1038/s41467-023-42333-z (PMC10579325; doi:10.1038/s41467-023-42333-z)
Supplement: Supplementary file 1 — Supplementary Information [file 41467_2023_42333_MOESM1_ESM.pdf]

Supplementary Information for

A Biocompatible Electrolyte Enables Highly Reversible Zn Anode for Zinc Ion Battery

Guanjie Li [1], Zihan Zhao [1, 2], Shilin Zhang [1, *], Liang Sun [1], Mingnan Li [1], Jodie A. Yuwono [1], Jianfeng Mao [1], Junnan Hao [1], Jitraporn (Pimm) Vongsvivut [3], Lidan Xing [4], Chun-Xia Zhao [1], Zaiping Guo [1, *]

Affiliations

[1] School of Chemical Engineering, Faculty of Sciences, Engineering and Technology, The University of Adelaide, SA 5005, Australia

[2] Department of Dermatology of Shanghai Skin Disease Hospital, Institute of Psoriasis, Tongji University School of Medicine, Shanghai 200443, China

[3] Infrared Microspectroscopy (IRM) Beamline, ANSTO–Australian Synchrotron, 800 Blackburn Road, Clayton, VIC 3168, Australia.

[4] School of Chemistry, South China Normal University, Guangzhou 510006, China

Email address: shilin.zhang01@adelaide.edu.au; zaiping.guo@adelaide.edu.au

Supplementary Figure S1

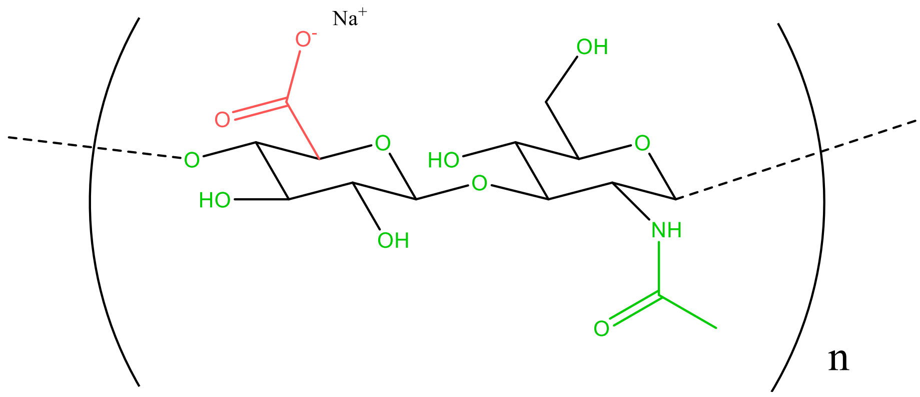

Supplementary Figure S1. Molecular structure of the HA.

Supplementary Figure S2

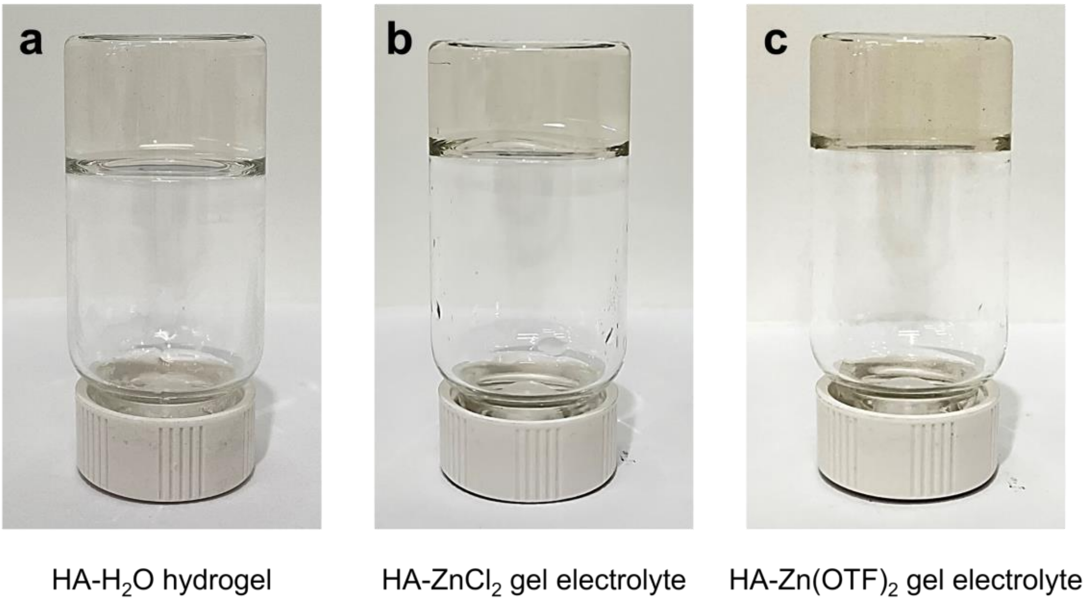

Supplementary Figure S2. Optical photos of gel electrolytes. (a) the HA-H_2O hydrogel, (b) the HA-$ZnCl_2$ gel electrolyte and (c) the HA-$Zn(OTF)_2$ gel electrolyte.

Supplementary Figure S3

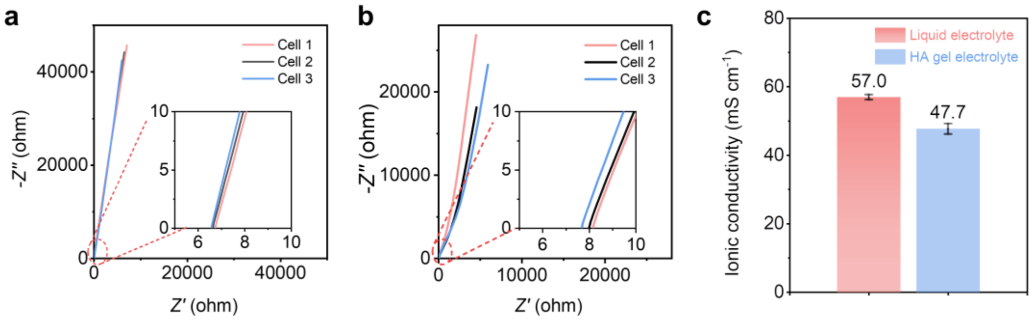

Supplementary Figure S3. Ionic conductivity of electrolytes. Nyquist plots of Ti//Ti symmetrical cells: (a) using the 2M $ZnSO_4$ liquid electrolyte and (b) the HA gel electrolyte. (c) Diagram of comparisons of ionic conductivity between the 2M $ZnSO_4$ liquid electrolyte and the HA gel electrolyte. The error bars represent the standard deviation of the values.

Supplementary Table S1. The comparison of ionic conductivity with previously reported gel electrolytes. Abbreviations of gel electrolytes are as follows: Hyaluronic acid (HA), poly(ethylene glycol) (PEG), poly(acrylamide-co-[2-(methacryloyloxy)ethyl]dimethyl-(3-sulfopropyl)) (PASHE), carboxyl-grafted polyvinyl alcohol and xanthan gum (PSX), poly(3-(1-vinyl-3-imidazolio) propanesulfonate) (PVIPS), poly-2-Acrylamido-2-methylpropanesulfonic/polyacrylamide (PAMPS-PAAM), polyacrylamide-poly (ethylene glycol) diacrylate-carboxymethyl cellulose (PMC), poly 2-acrylamido-2-methyl-1-propane sulfonate zinc (PAMPSZn), iota-carrageenan (IC), polyacrylamide (ZS/GL/AN), sorbitol-modified cellulose (Sor-Cel).

Gel electrolytes	Salt	Solvent	Ionic conductivity (mS cm^{-1})	Ref.
**HA**	2M $ZnSO_4$	H_2O	47.7	This work
**PEG**	$Zn(ClO_4)_2$	/	12.6	1
**PASHE**	1M $ZnSO_4$	H_2O	32.9	2
**PSX**	2M $ZnSO_4$	H_2O	18.9	3
**PVIPS**	2M $ZnSO_4$ + 0.1M $MnSO_4$	H_2O	21.9	4
**PAMPS-PAAM**	$ZnSO_4$	DMSO	21.6	5
**PMC**	2M $ZnSO_4$	H_2O	30.2	6
**PAMPSZn**	$Zn(OH)_2$•$2ZnCO_3$	H_2O	15.6	7
**IC**	2M $ZnSO_4$	H_2O	43.0	8
**ZS/GL/AN**	3M $ZnSO_4$	H_2O	13.9	9
**Sor-Cel**	16M $ZnCl_2$ + 0.6M $CaCl_2$	H_2O	35.4	10

Supplementary Figure S4

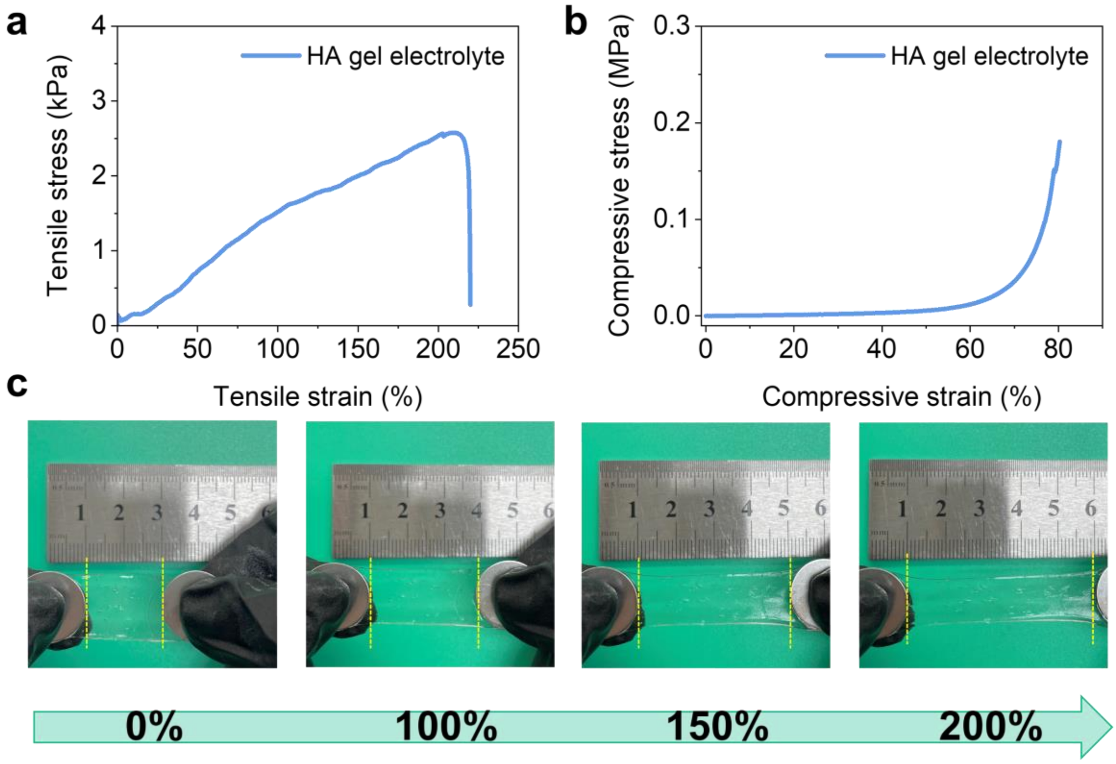

Supplementary Figure S4. Mechanical strength of the HA gel electrolyte. (a) Tensile and (b) compressive stress–strain curves of the HA gel electrolyte. (c) Optical photos showing the stretching process of the HA gel electrolyte.

Supplementary Figure S5

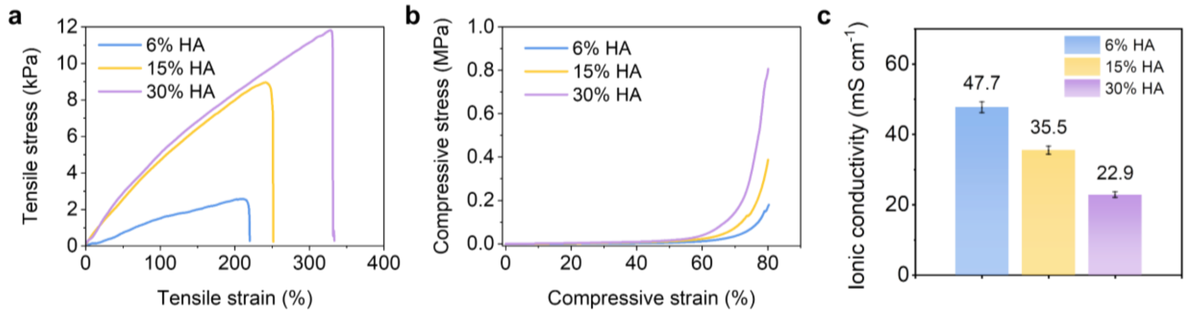

Supplementary Figure S5. Mechanical strength of HA gel electrolytes with different water contents. (a) Tensile and (b) compressive stress–strain curves and (c) ionic conductivity of HA gel electrolytes. The error bars represent the standard deviation of the values.

Supplementary Table S2. The comparison of tensile strength and compressive strength with previously reported gel electrolytes. Abbreviations of gel electrolytes are as follows: Hyaluronic acid (HA), poly(acrylamide-co-[2-(methacryloyloxy)ethyl]dimethyl-(3-sulfopropyl)) (PASHE), poly(3-(1-vinyl-3-imidazolio) propanesulfonate) (PVIPS), polyacrylamide-poly (ethylene glycol) diacrylate-carboxymethyl cellulose (PMC), iota-carrageenan (IC), polyacrylamide (ZS/GL/AN), sorbitol-modified cellulose (Sor-Cel).

Gel electrolytes	Elongation-at-break (%)	Compressive strength (MPa)	Compressive strain (%)	Ref.
**HA-6%**	220	0.18	80%	This work
**HA-15%**	251	0.39	80%	This work
**HA-30%**	333	0.81	80%	This work
**PASHE**	315	0.05	60%	[2]
**PVIPS**	62	/	/	[4]
**PMC**	100	/	/	[6]
**IC**	128	/	/	[8]
**ZS/GL/AN**	350	/	/	[9]
**Sor-Cel**	303	0.085	50%	[10]

Supplementary Figure S6

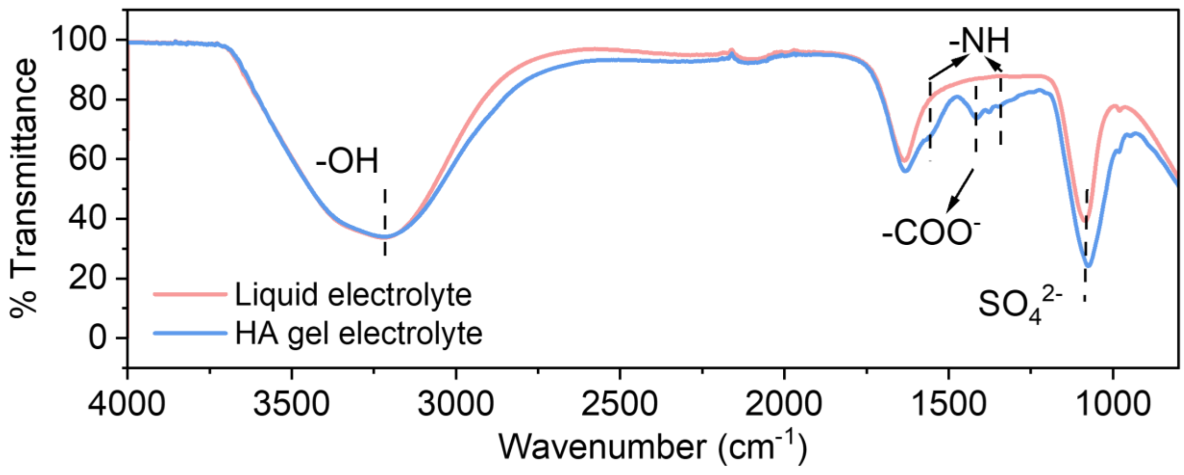

Supplementary Figure S6. Full range FTIR spectra of liquid and HA gel electrolytes.

Supplementary Figure S7

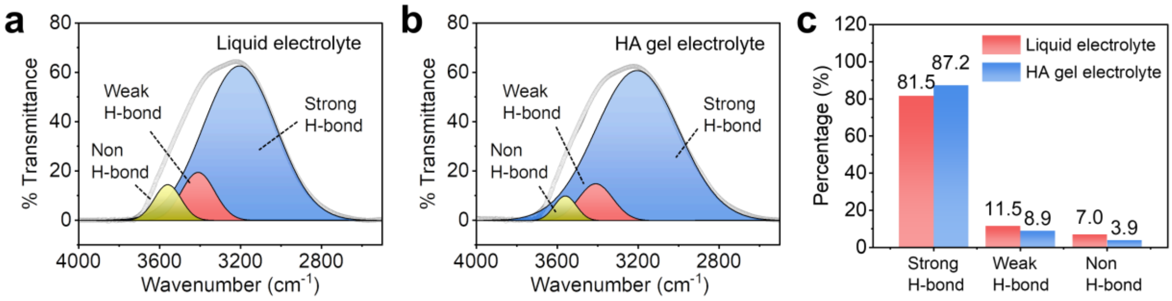

Supplementary Figure S7. Comparison of H-bonds in different electrolytes. FTIR spectra and related curve fitting results in the range of 2400–4000 cm^{-1} observed in the (a) liquid electrolyte and (b) HA gel electrolyte. (c) Ratios of different hydrogen bonds calculated from fitting results of FTIR spectra in (a) and (b).

Supplementary Figure S8

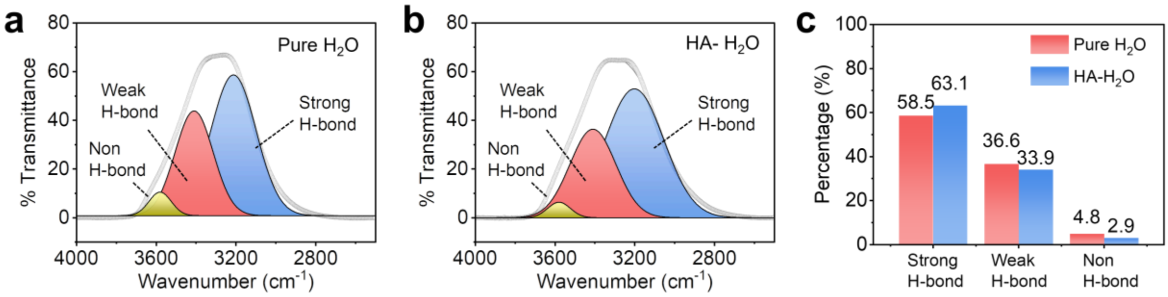

Supplementary Figure S8. Comparison of H-bonds in H_2O and HA gel. FTIR spectra and related curve fitting results in the range of 2400–4000 cm^{-1} observed in (a) pure water and (b) HA-H_2O gel electrolyte. (c) Ratios of different hydrogen bonds calculated from fitting results of FTIR spectra in (a) and (b).

Supplementary Figure S9

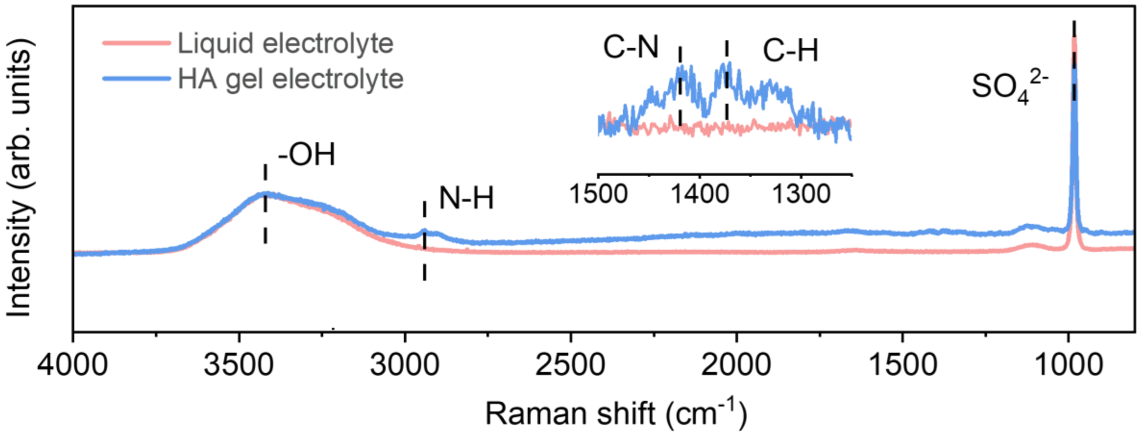

Supplementary Figure S9. Full range Raman spectra of liquid and HA gel electrolytes.

Supplementary Figure S10

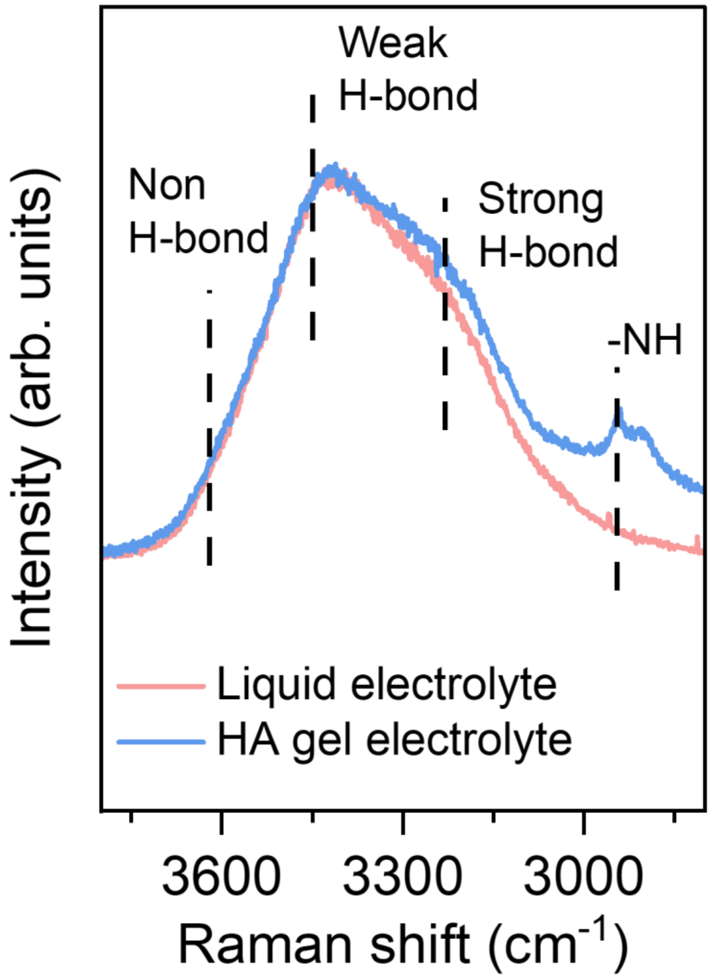

Supplementary Figure S10. Raman spectra of liquid and HA gel electrolytes in selected range.

Supplementary Figure S11

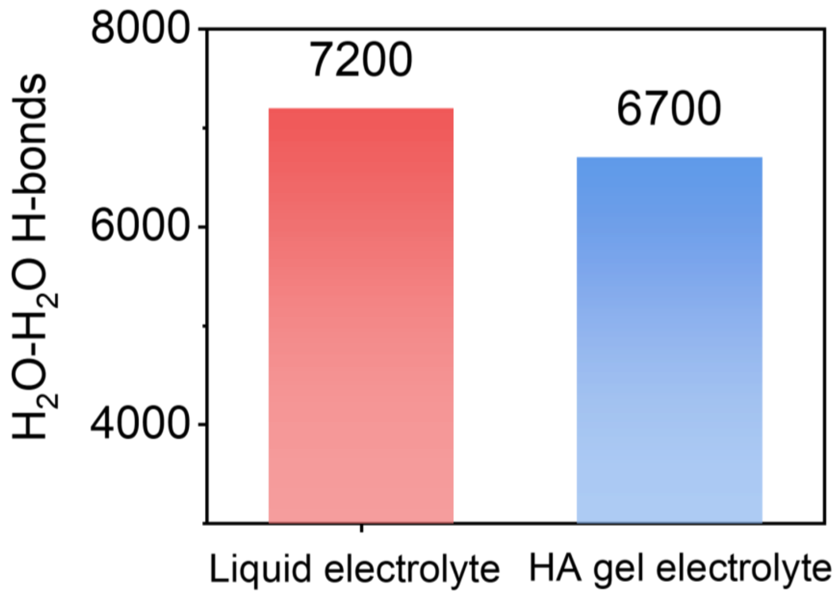

Supplementary Figure S11. The amount of hydrogen bonds between H_2O and H_2O in liquid and HA gel electrolytes by using MD simulation.

Supplementary Table S3. Comparison of HER potentials on various working electrodes. The HER overpotential is obtained from previous reports.[11-14]

Electrode	HER overpotential (V)		
	j=1000 mA cm^{-2}	j=10 mA cm^{-2}	j=1 mA cm^{-2}
**Pt**	0.44	0.39	0.09
**Zn**	1.24	0.75	0.72
**Ti**	0.82	0.6	/
**Glassy carbon**	1.13	/	/
**Graphite**	1.03	0.76	0.47
**Stainless steel**	/	0.42	/

Supplementary Figure S12

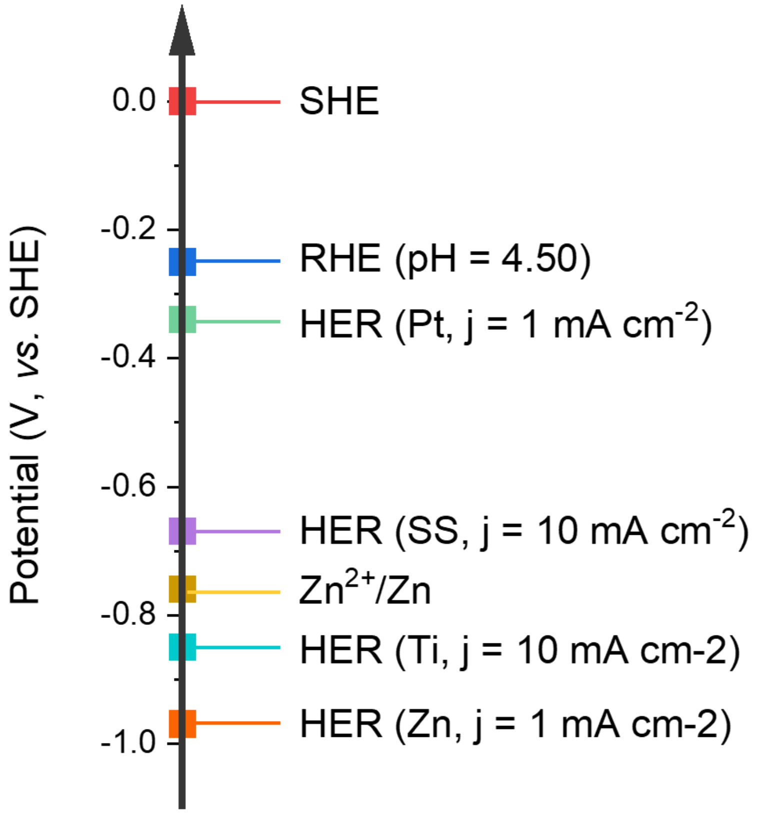

Supplementary Figure S12. The Zn^{2+}/Zn electrode potential and HER potentials on various working electrodes.

Supplementary Figure S13

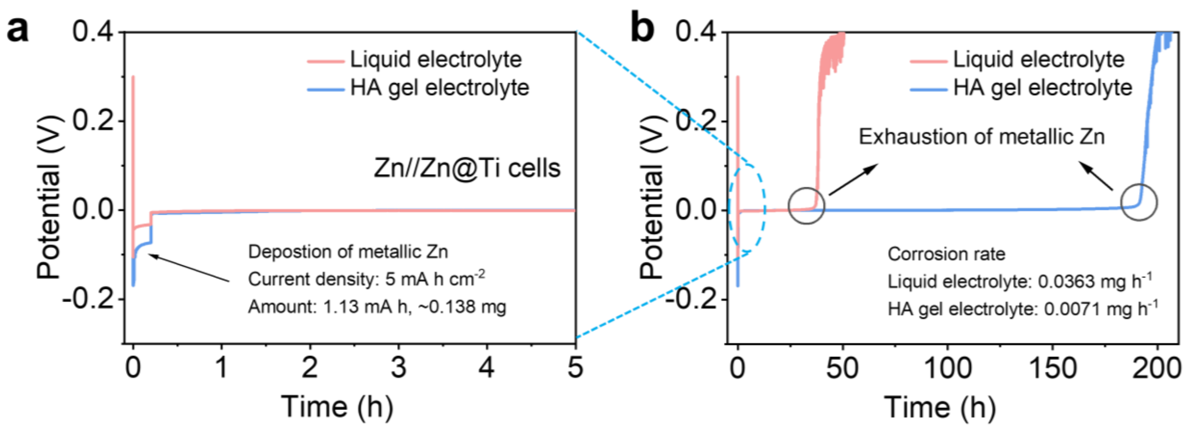

Supplementary Figure S13. Corrosion rate of Zn anodes in two electrolytes. Voltage-time curves for Zn@Ti electrodes immersed in the liquid electrolyte and the HA gel electrolyte. An amount of metallic Zn, 1.13 mAh, was deposited on Ti-foil to prepare the Zn@Ti electrode. Profiles were recorded for (a) the initial 5 hours and (b) 220 hours.

Supplementary Figure S14

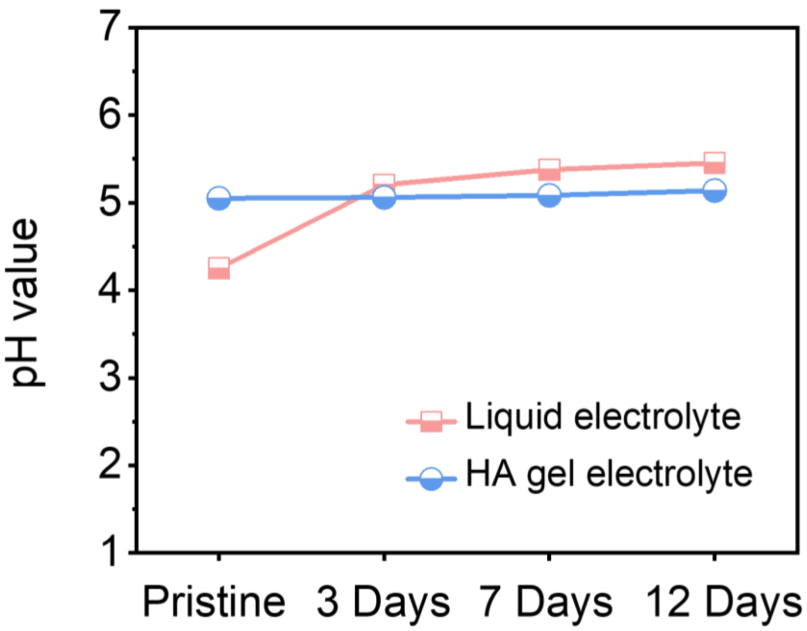

Supplementary Figure S14. The pH variation of liquid and HA gel electrolytes with Zn anodes immersed in each electrolyte for different days.

Supplementary Figure S15

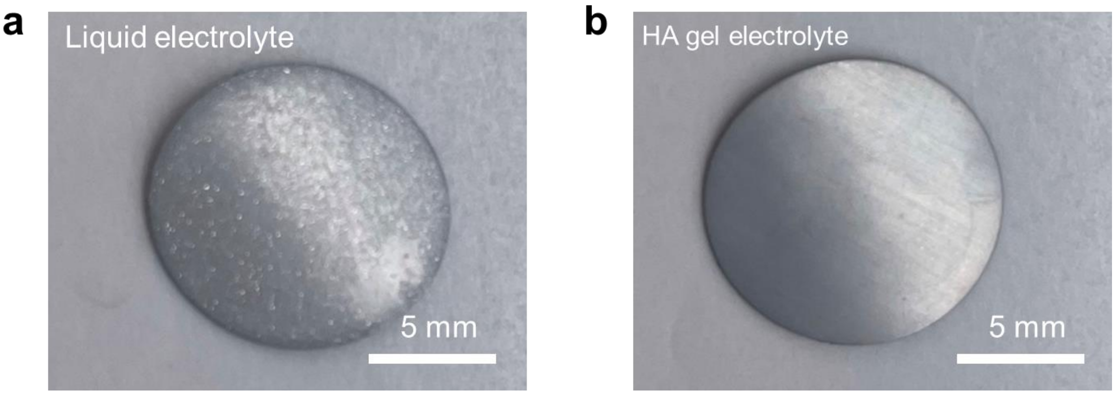

Supplementary Figure S15. Optical photos of Zn metals after storage in two electrolytes. (a) The liquid electrolyte and (b) the HA gel electrolyte for 12 days.

Supplementary Figure S16

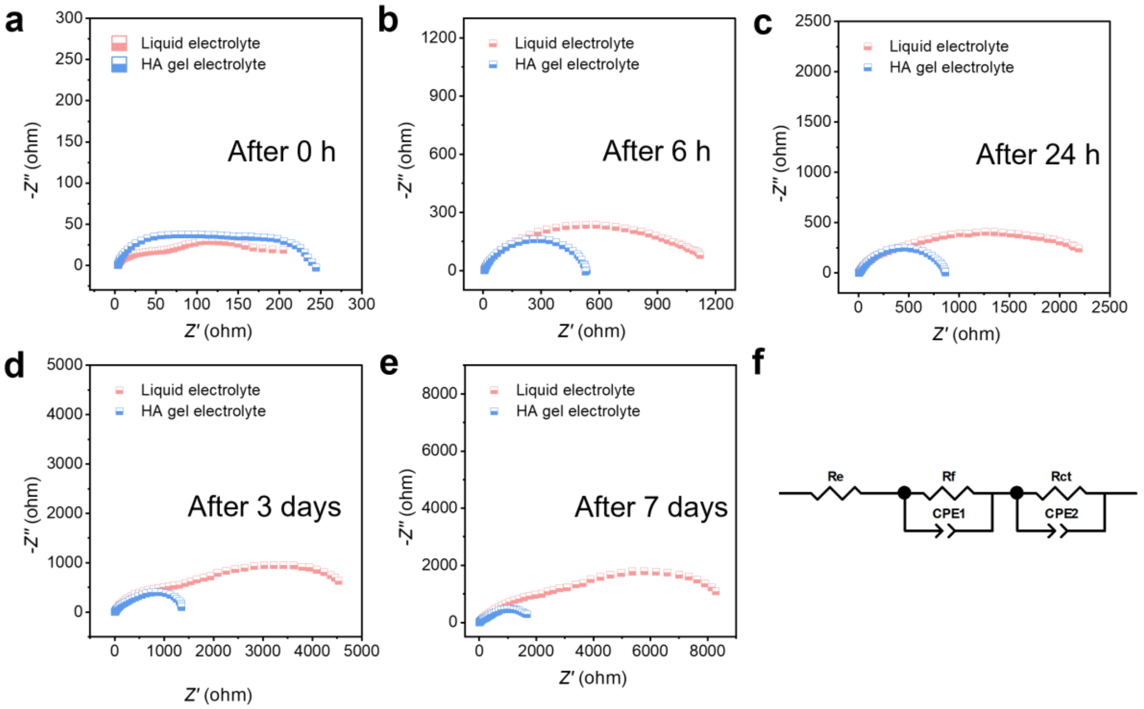

Supplementary Figure S16. Impedance variation of Zn anodes during storage. Nyquist plots of Zn//Zn symmetric cells when immersing in liquid and HA gel electrolytes for (a) 0h, (b) 6h, (c) 24h, (d) 3 days and (e) 7days, and (f) corresponding fitting equivalent circuit.

Supplementary Figure S17

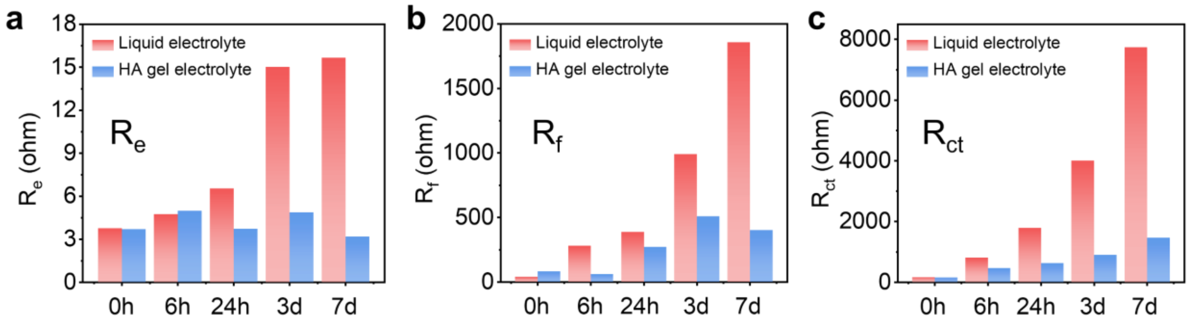

Supplementary Figure S17. Characterisation of interfacial impedance of Zn//Zn symmetric cells during the immersion. (a) R_e, (b) R_f and (c) R_{ct}.

Supplementary Table S4. Fitting results of interfacial impedance of Zn//Zn symmetric cells when immersing in liquid and HA gel electrolytes.

**Time**	***R_e* (Ω)**		***R_f* (Ω)**		***R_{ct}* (Ω)**	
	**Liquid electrolyte**	**HA gel electrolyte**	**Liquid electrolyte**	**HA gel electrolyte**	**Liquid electrolyte**	**HA gel electrolyte**
**0h**	3.8	3.7	38.6	82.3	170.1	153.8
**6h**	4.8	5.0	280.0	60.7	811.3	470.9
**24h**	6.5	3.7	387.3	272.0	1789.0	633.6
**3d**	15.0	4.9	989.6	508.2	4003.0	901.5
**7d**	15.7	3.2	1855.0	402.7	7740.0	1463.0

Supplementary Figure S18

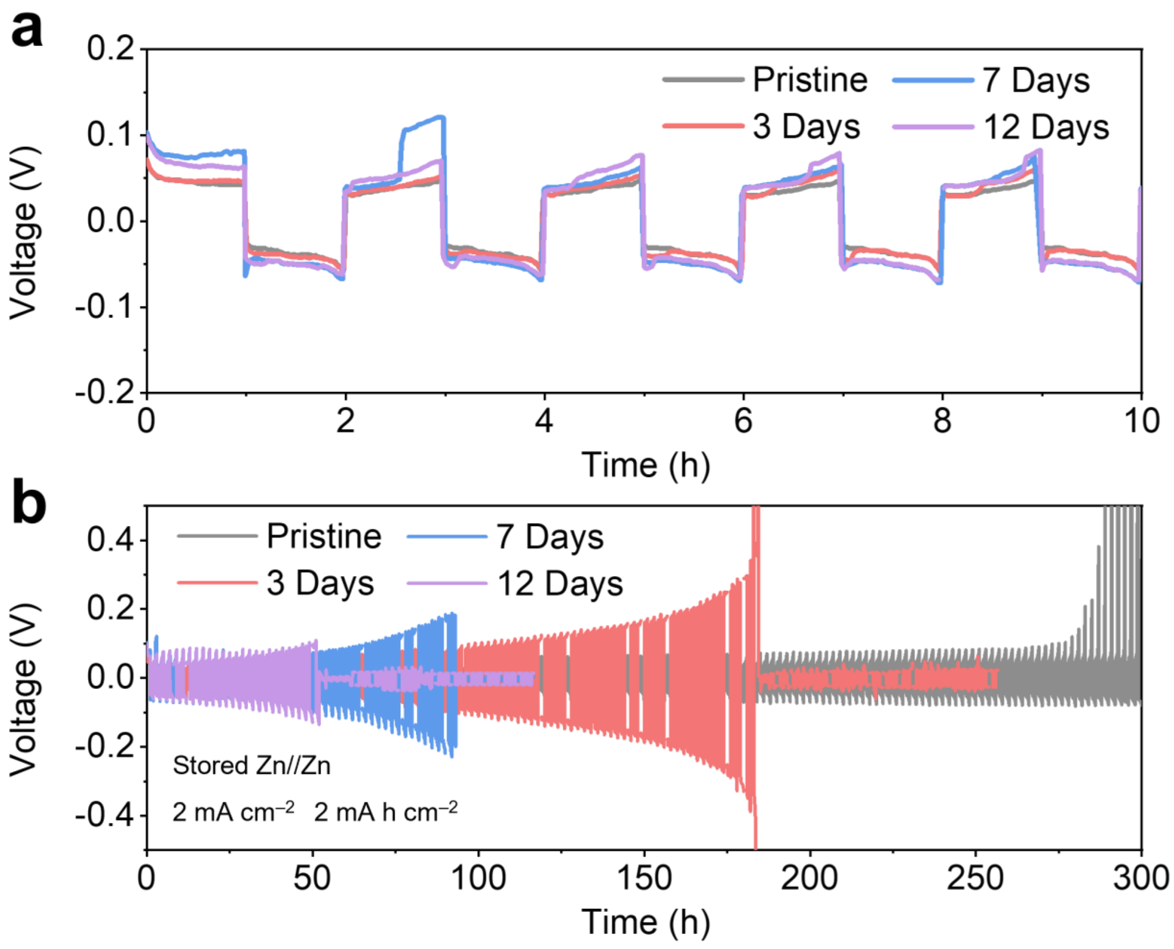

Supplementary Figure S18. Time-voltage profiles of aged Zn//Zn symmetric cells in liquid electrolyte at a current density of 2 mA cm^{-2}, with a total capacity of 2 mA h cm^{-2}: Profiles were recorded for (a) the initial 10 hours and (b) 300 hours. After storage in the liquid electrolyte for 12 days, the Zn anode exhibited rapid failure, lasting only for 50 hours. This performance is worse than that of a pristine Zn anode, which can last at least 250 hours.

Supplementary Figure S19

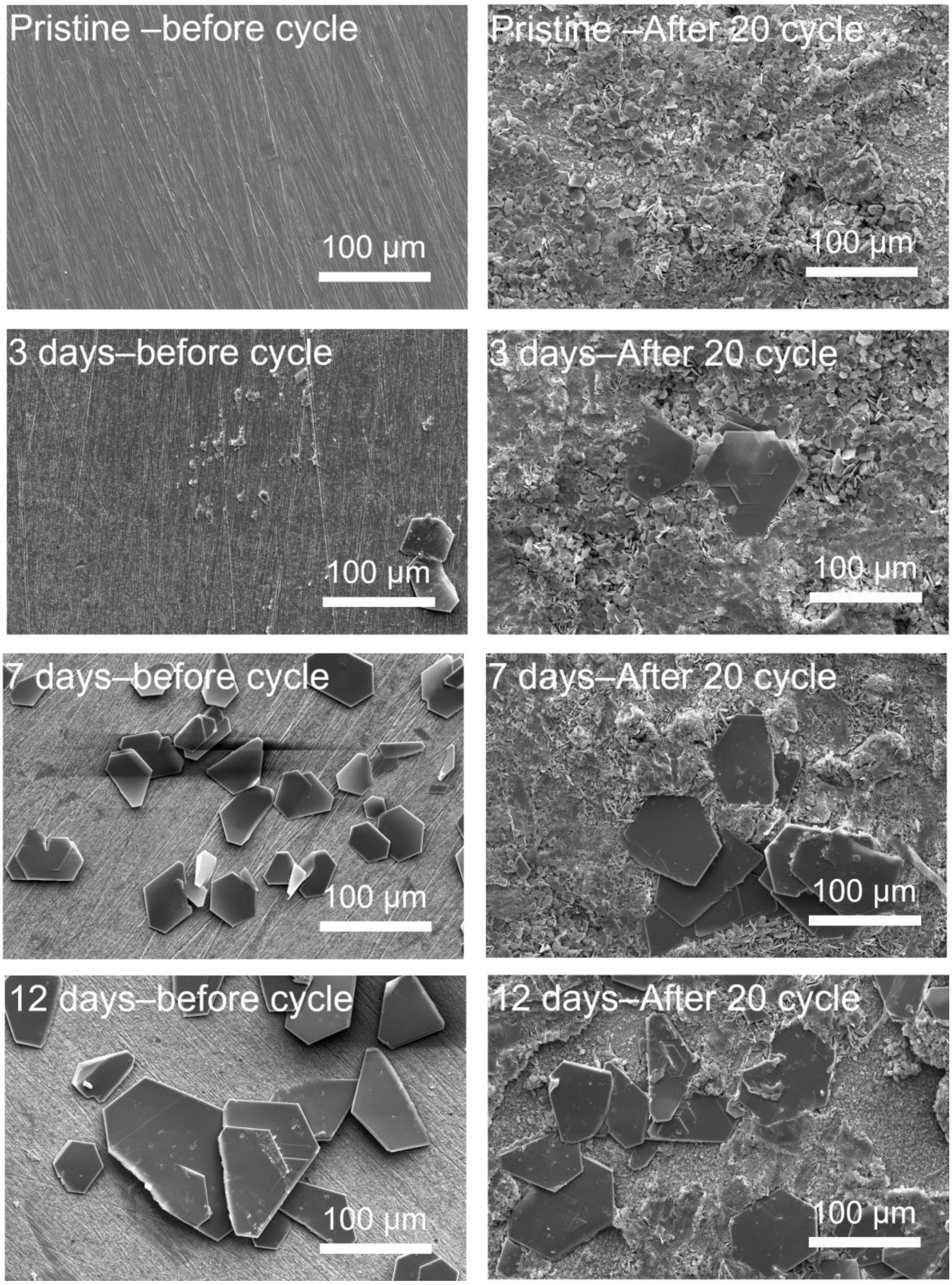

Supplementary Figure S19. SEM images of Zn anodes in aged Zn//Zn symmetric cells taken before and after 20 cycles at a current density of 2 mA cm^{-2}, with a total capacity of 2 mA h cm^{-2} in the liquid electrolyte.

Supplementary Figure S20

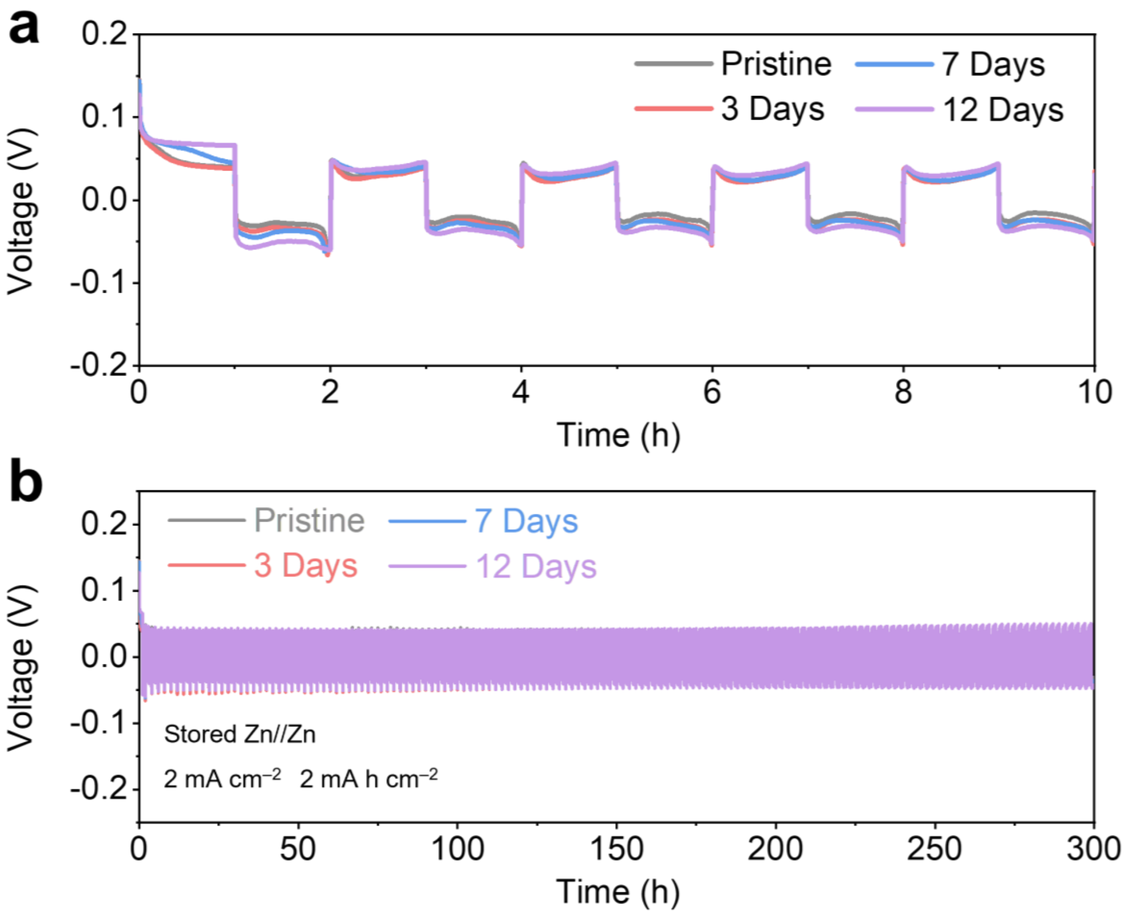

Supplementary Figure S20. Time-voltage profiles of aged Zn//Zn symmetric cells in the HA gel electrolyte at a current density of 2 mA cm^{-2}, with a total capacity of 2 mA h cm^{-2}: Profiles were recorded for (a) the initial 10 hours and (b) 300 hours.

Supplementary Figure S21

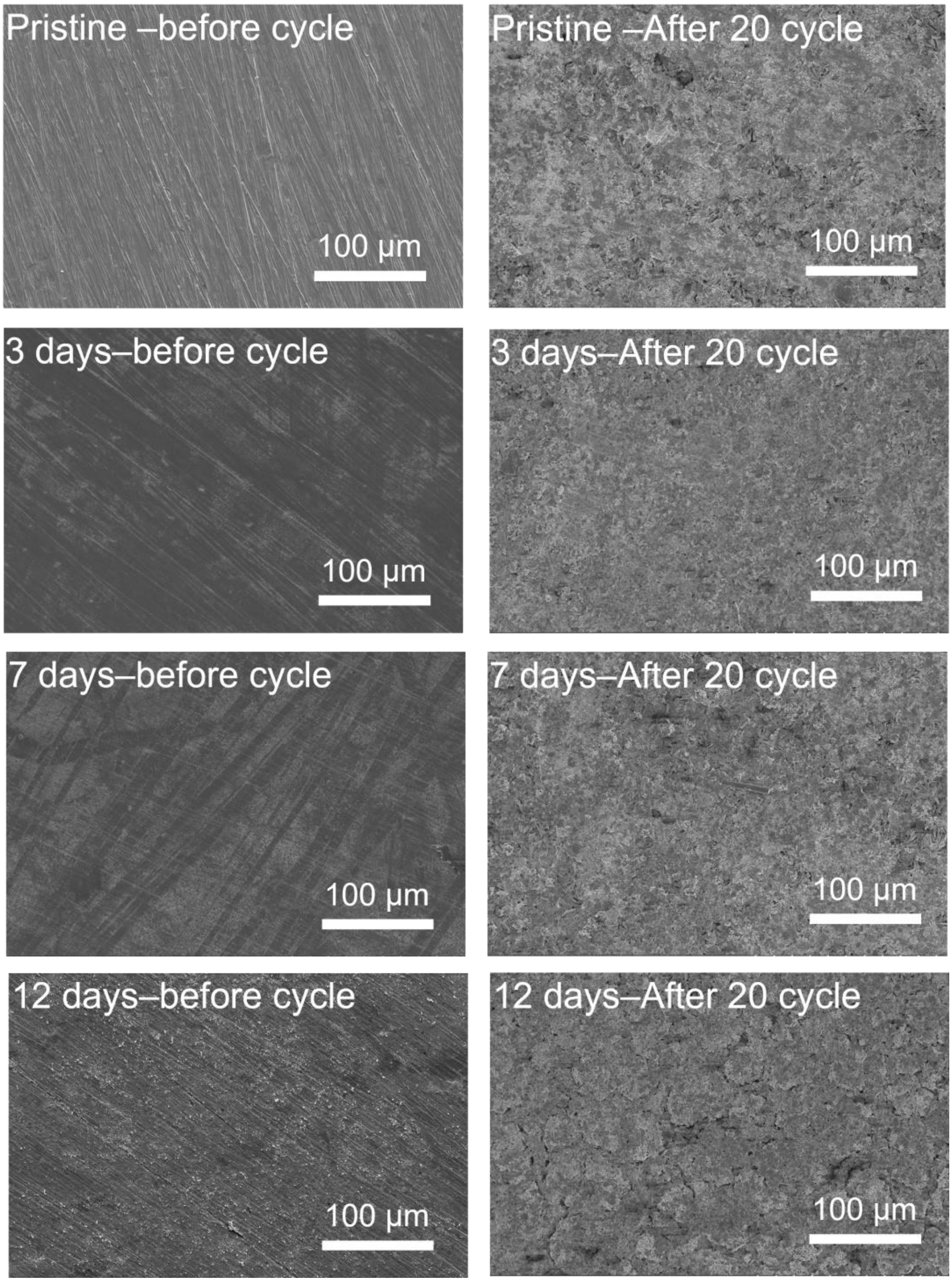

Supplementary Figure S21. SEM images of Zn anodes in aged Zn//Zn symmetric cells taken before and after 20 cycles at a current density of 2 mA cm^{-2}, with a total capacity of 2 mA h cm^{-2} in the HA gel electrolyte.

Supplementary Figure S22

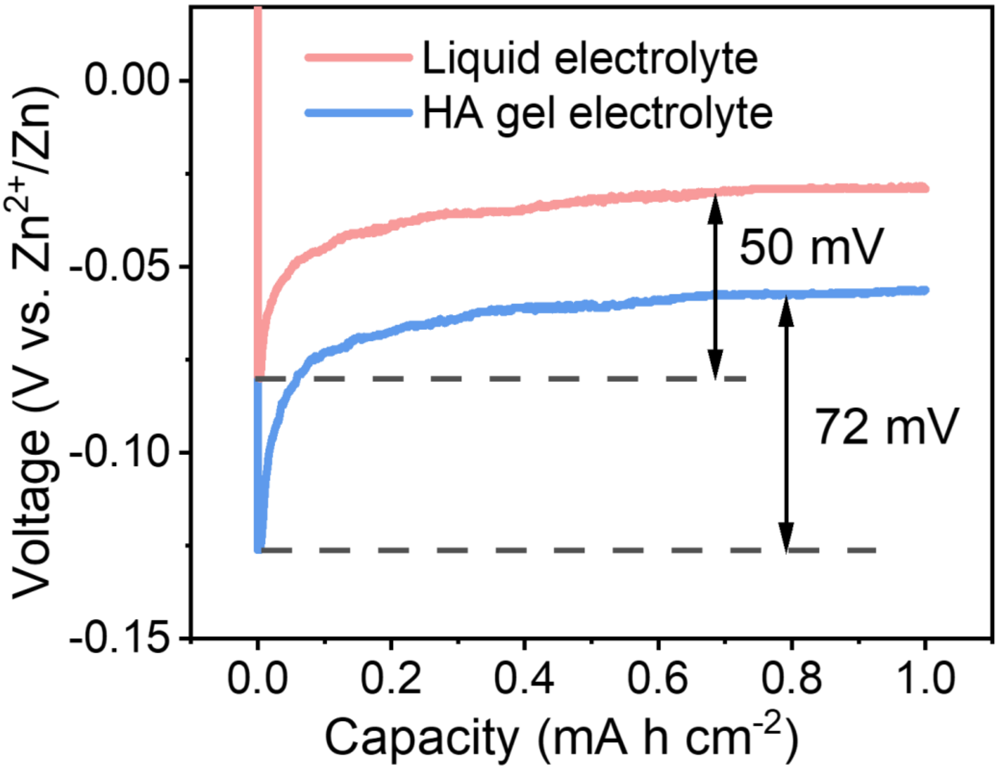

Supplementary Figure S22. Nucleation overpotential observed on Zn//Cu cells at a current density of 1 mA cm^{-2}.

Supplementary Figure S23

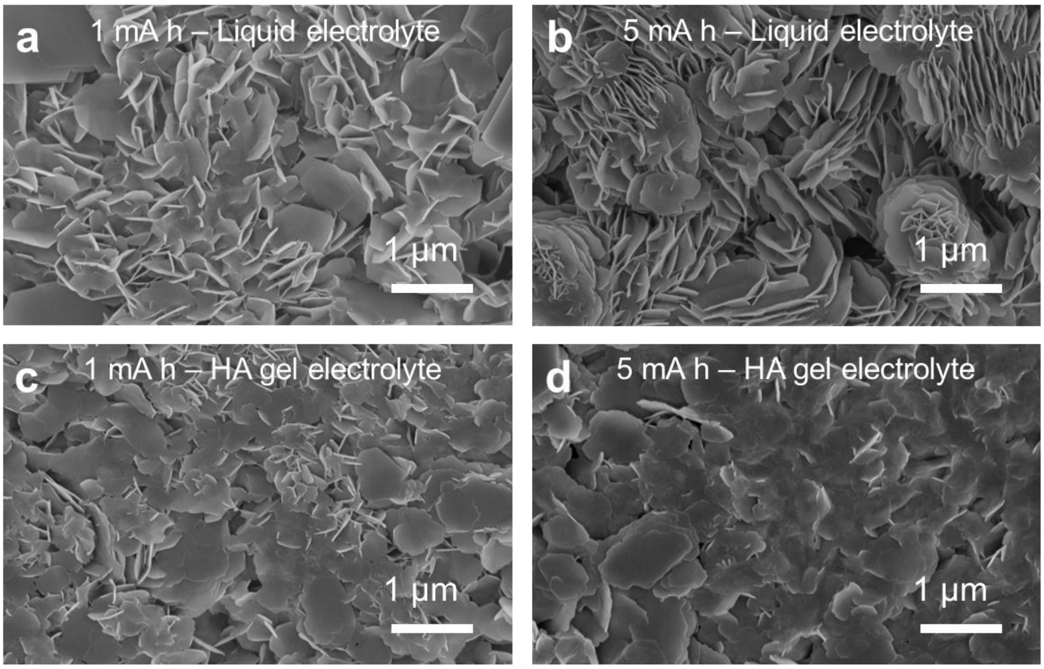

Supplementary Figure S23. Morphology of deposited Zn. SEM images of plating Zn on Cu current collectors at a consistent current density of 1 mA cm^{-2}, with different areal capacities in (a, b) the liquid electrolyte and (c, d) the HA gel electrolyte.

Supplementary Figure S24

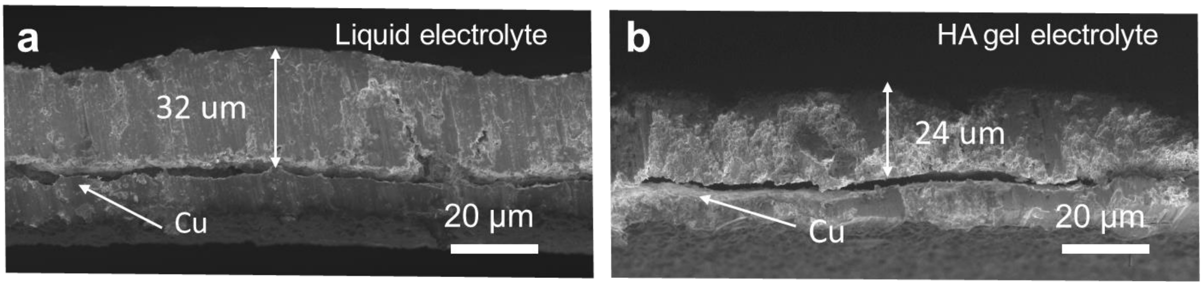

Supplementary Figure S24. Thickness of deposited Zn layers. Cross-section SEM images of the Zn layer after plating with a total capacity of 10 mA h cm^{-2} on Cu current collectors in (a) the liquid electrolyte and (b) the HA gel electrolyte.

Supplementary Figure S25

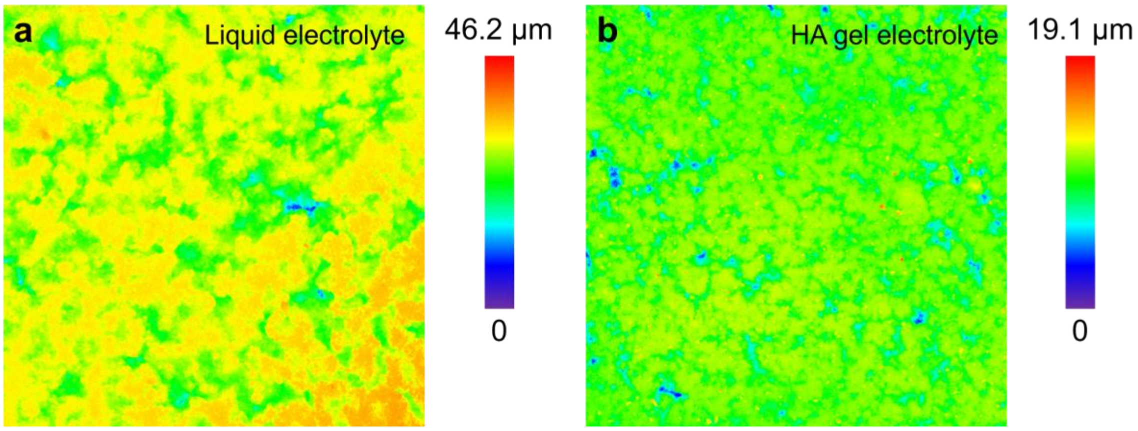

Supplementary Figure S25. Two-dimensional confocal laser microscopy images of the Zn layer after plating with a capacity of 10 mA h cm^{-2} on Cu current collectors in (a) the liquid electrolyte and (b) the HA gel electrolyte.

Supplementary Figure S26

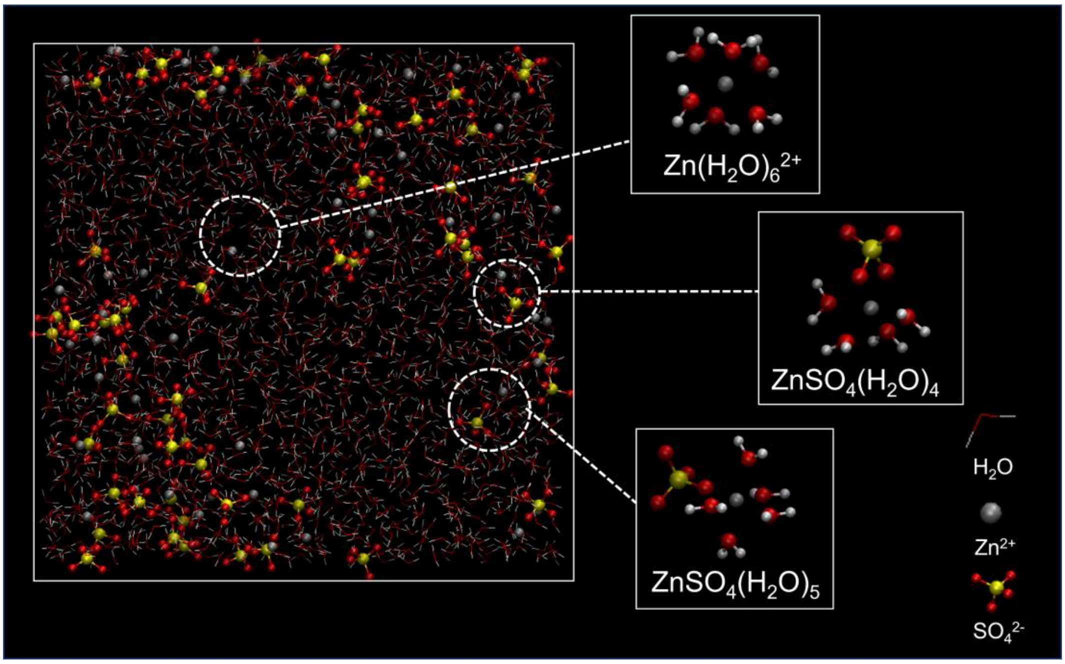

Supplementary Figure S26. Snapshots of the MD simulation model of the liquid electrolytes. Atom colours: Zn (grey), O (red), H (white), S (yellow).

Supplementary Figure S27

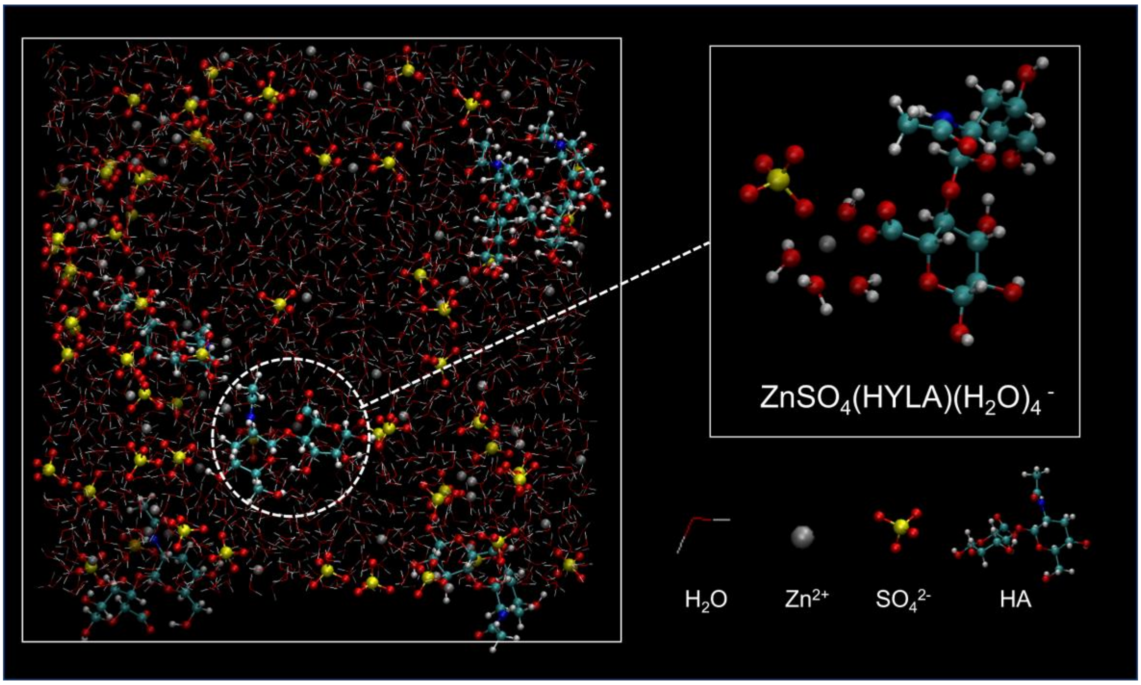

Supplementary Figure S27. Snapshots of the MD simulation model of the HA gel electrolytes. Atom colours: Zn (grey), O (red), H (white), S (yellow), C (indigo), N (blue).

Supplementary Figure S28

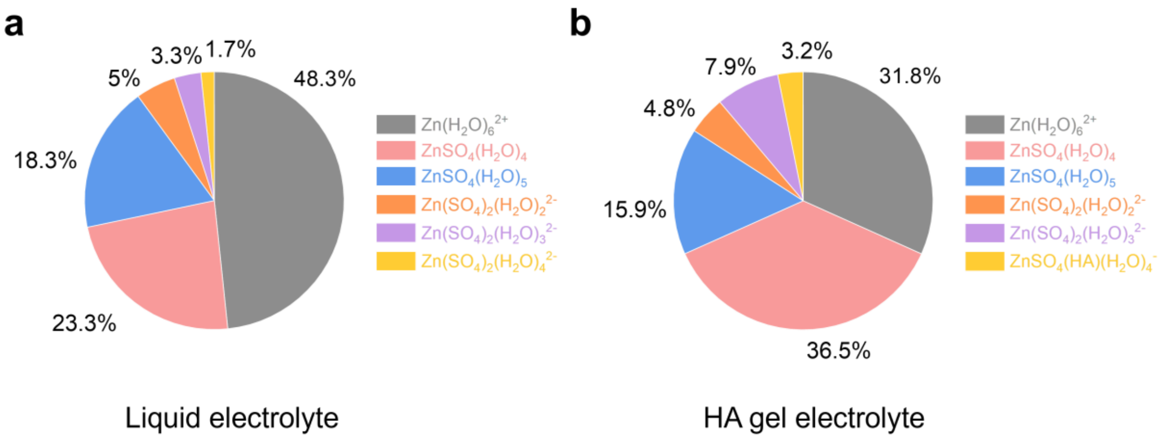

Supplementary Figure S28. The percentage of various Zn^{2+} solvation clusters in (a) the liquid and (b) the HA gel electrolytes obtained from MD simulations.

Supplementary Figure 29

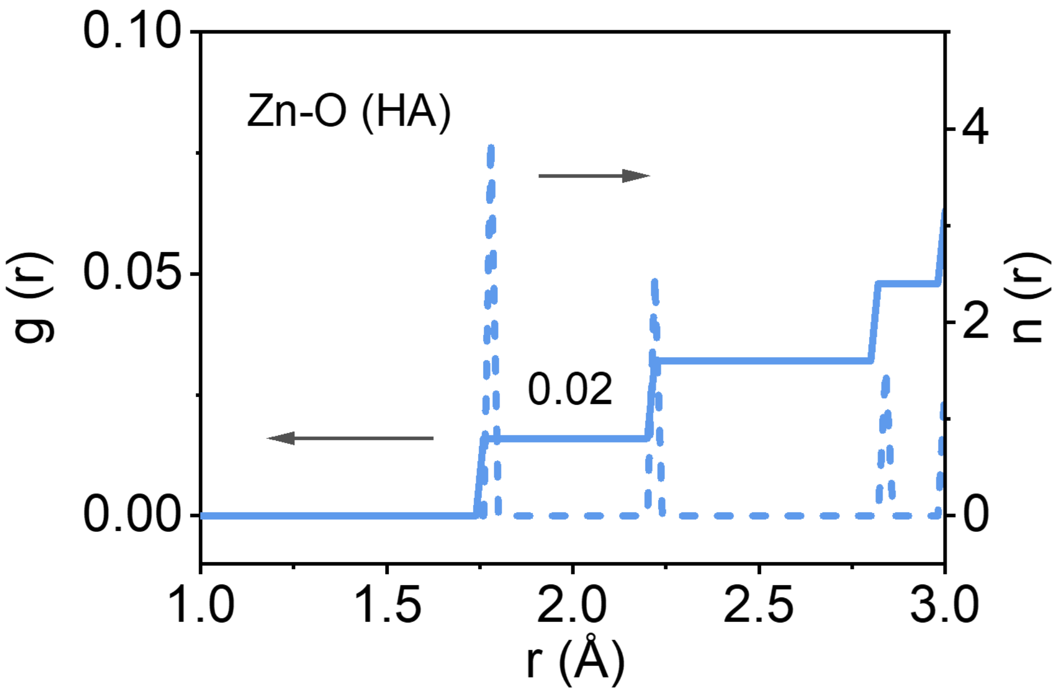

Supplementary Figure S29. The radial distribution function g(r) and coordination number n(r) of Zn^{2+}–O (HA) obtained from MD simulations in the HA gel electrolyte.

Supplementary Figure S30

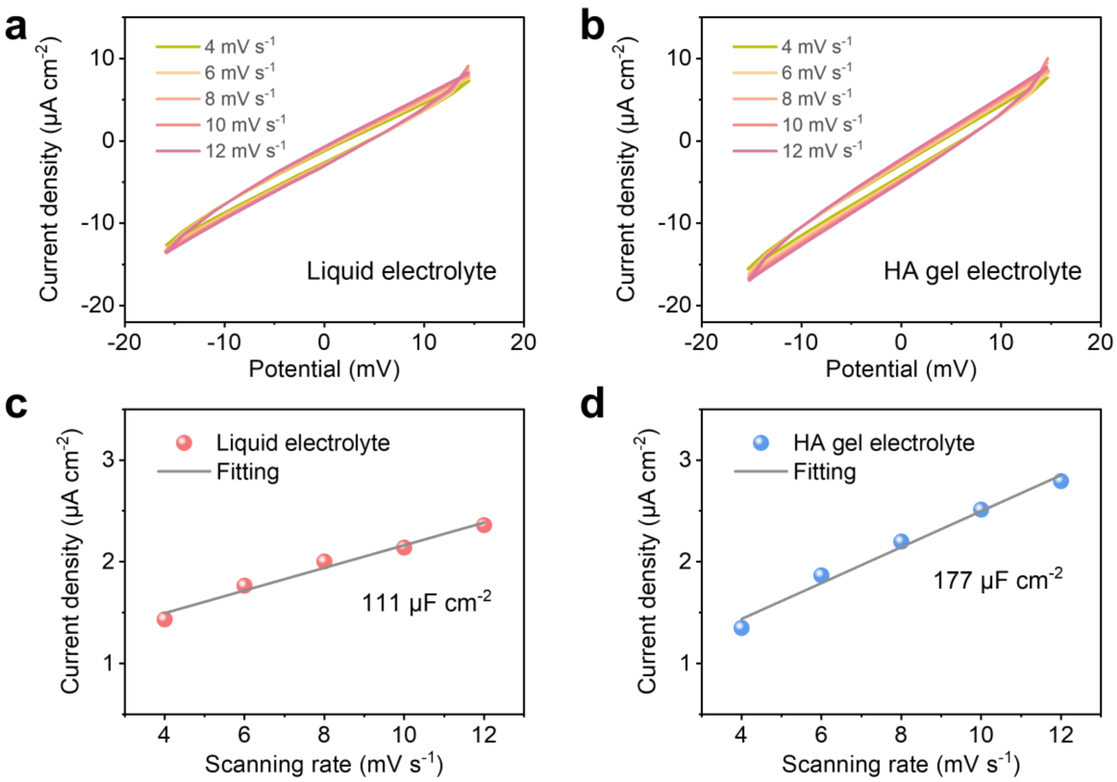

Supplementary Figure S30. Capacitance of electric double layer between Zn anodes and electrolytes. Cyclic voltammograms and current density-scanning rate fitting result of Zn//Zn symmetric cells using (a, c) the liquid electrolyte and (b, d) the HA gel electrolyte.

Supplementary Figure S31

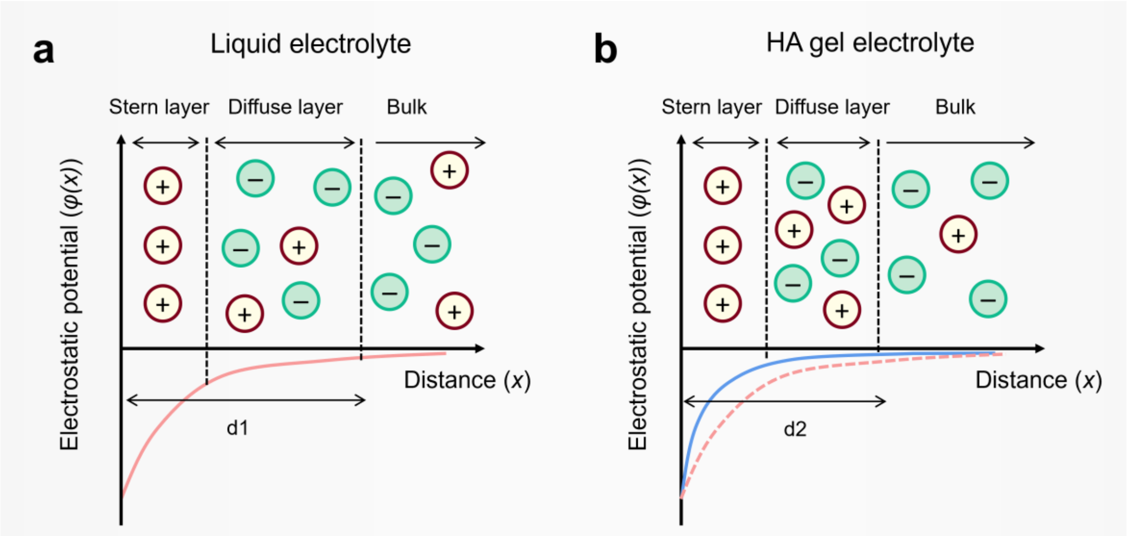

Supplementary Figure S31. The comparison of the electric double layer: (a) between the Zn anode and the liquid electrolyte; (b) between the Zn anode and the HA gel electrolyte. The relationship between electrostatic potential ($\varphi(x)$) and distance (x) follows the equation $\varphi(x) = \varphi_0 * \exp(-\kappa * x)$, where φ_0 is the potential at the surface of the electrode ($x = 0$), and κ the inverse of the Debye length.

Supplementary Figure S32

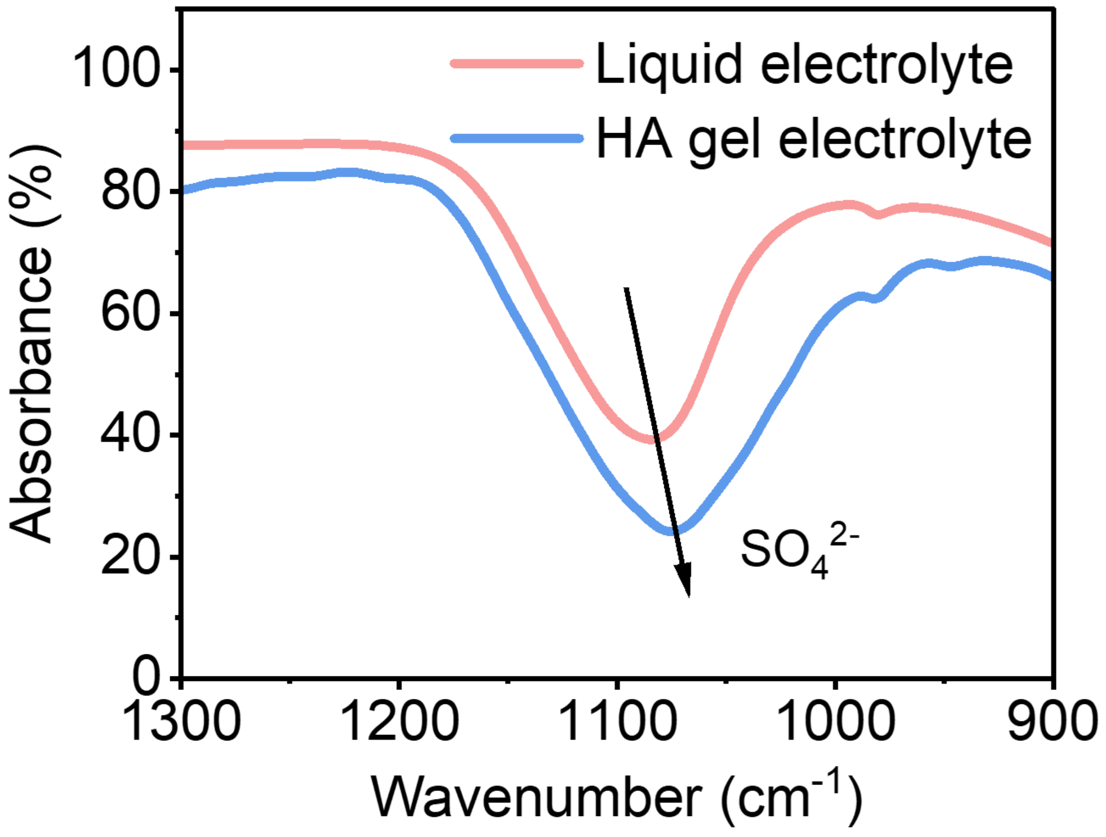

Supplementary Figure S32. FTIR spectra of liquid and HA gel electrolytes in a selected range.

Supplementary Figure S33

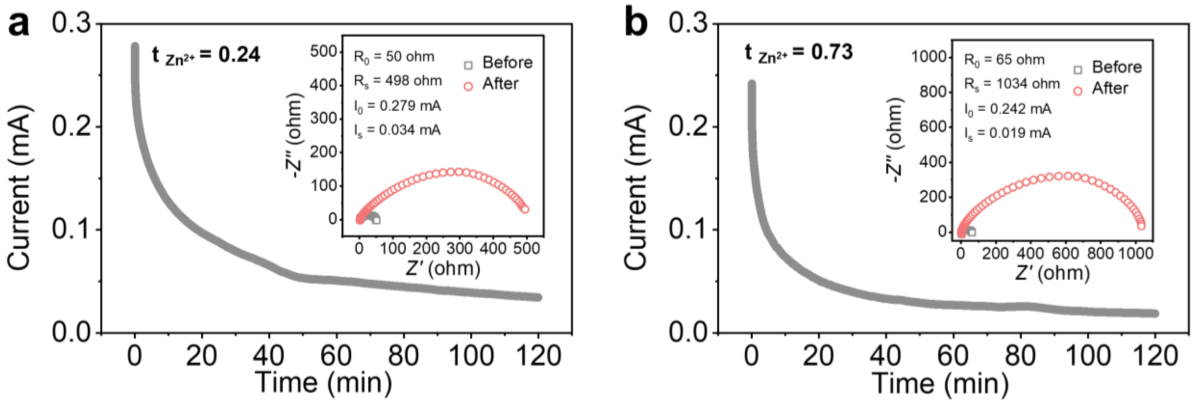

Supplementary Figure S33. Transference number of Zn^{2+}. Polarisation profile and Nyquist plots before and after polarization of Zn//Zn symmetric cells in (a) the liquid electrolyte and (b) the HA gel electrolyte.

Supplementary Figure S34

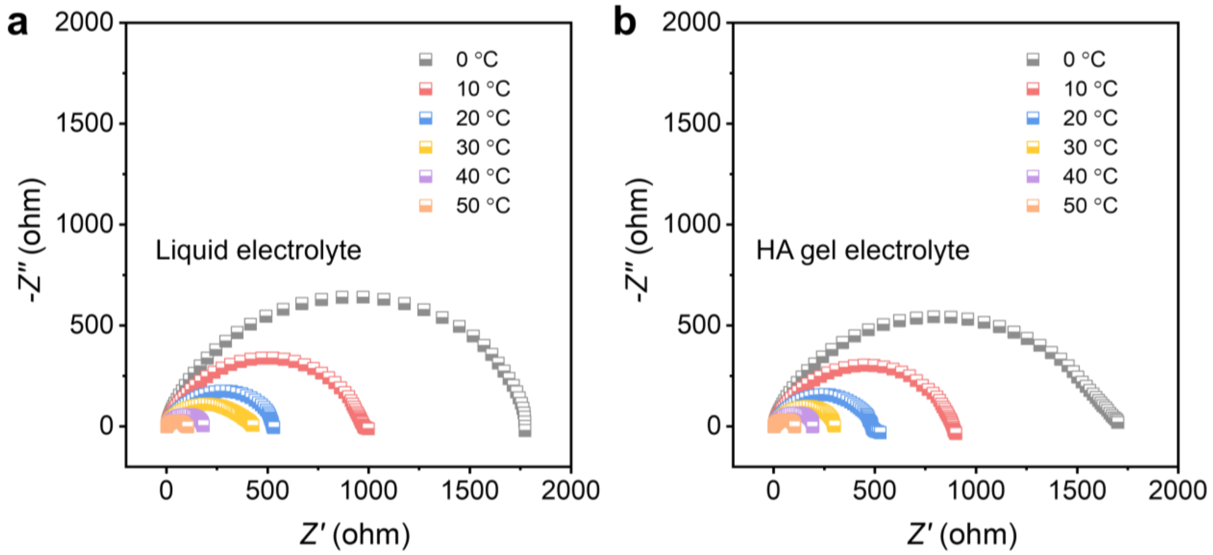

Supplementary Figure S34. Nyquist plots of Zn//Zn symmetric cells testing under various temperatures in (a) the liquid electrolyte and (b) the HA gel electrolyte.

Supplementary Figure S35

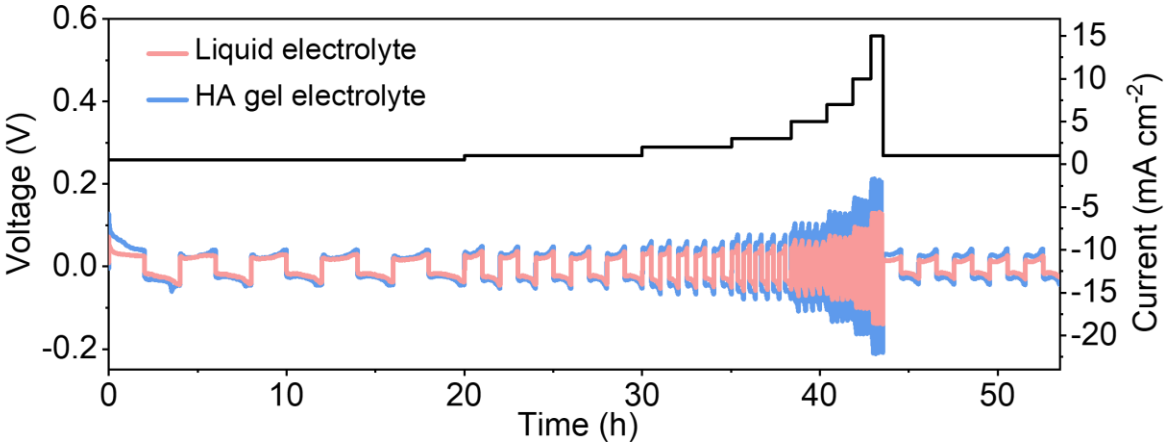

Supplementary Figure S35. Rate performance of Zn//Zn symmetric cells in liquid and HA gel electrolytes.

Supplementary Figure S36

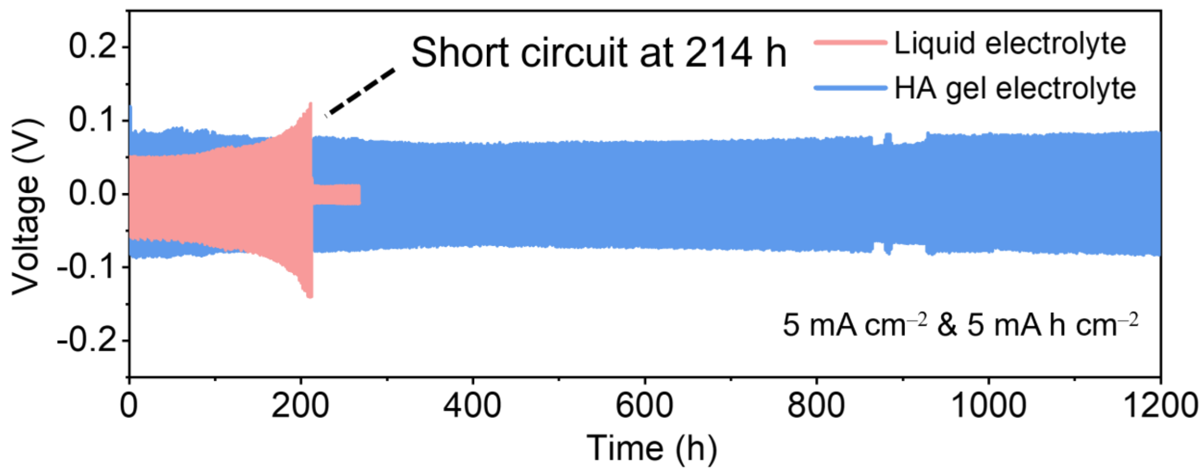

Supplementary Figure S36. Long-term cycle performance of Zn//Zn symmetric cells in liquid and HA gel electrolytes at a current density of 5 mA cm^{-2}, with a capacity of 5 mA h cm^{-2}. The accumulated capacities can be calculated to be 3 A h cm^{-2} (5 mA cm^{-2} ×1200 h/2, where 2 denotes the time to complete a charge-discharge cycle.)

Supplementary Figure S37

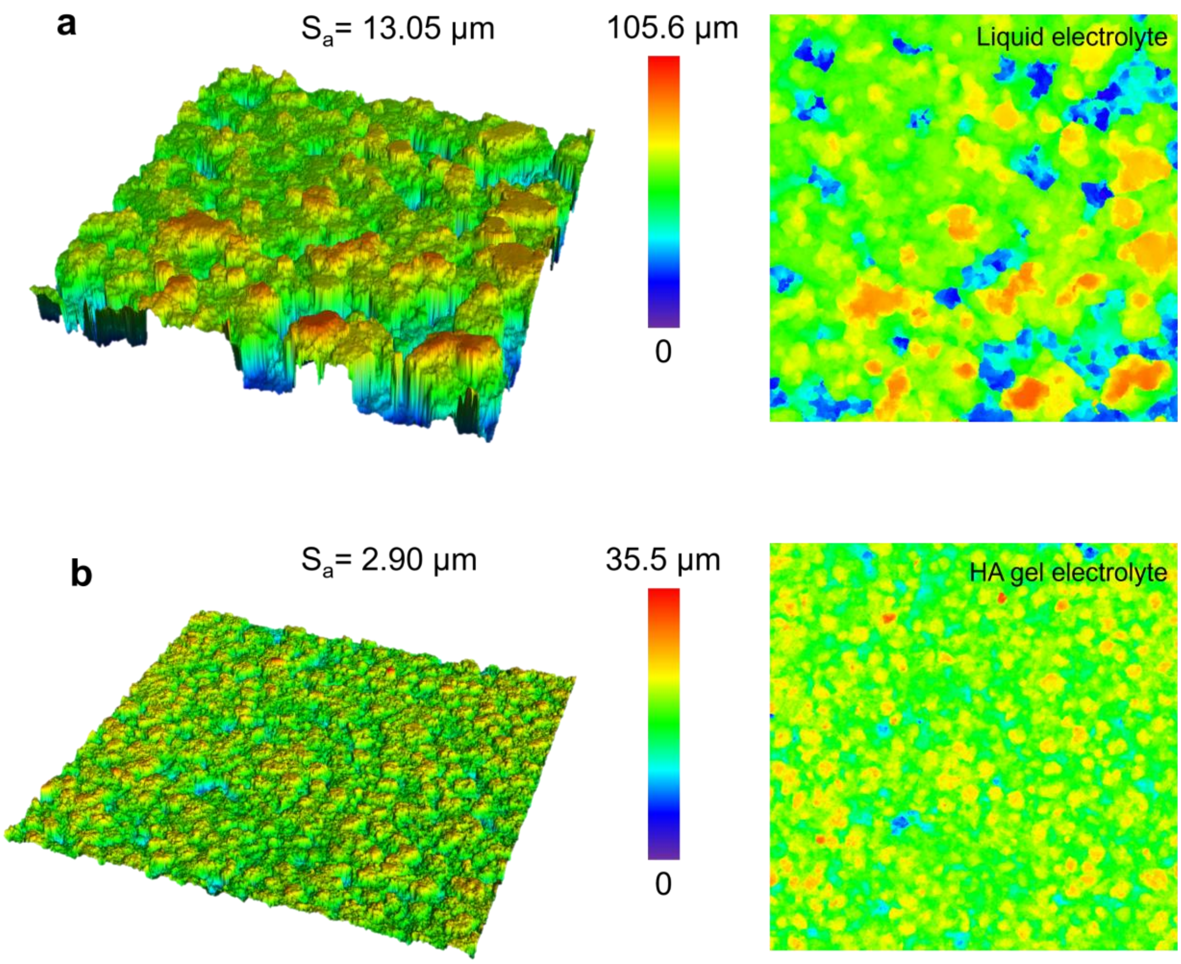

Supplementary Figure S37. Confocal laser microscopy images of Zn metal after 100 cycles in (a) the liquid electrolyte and (b) the HA gel electrolyte. *Sa:* arithmetic mean height.

Supplementary Figure S38

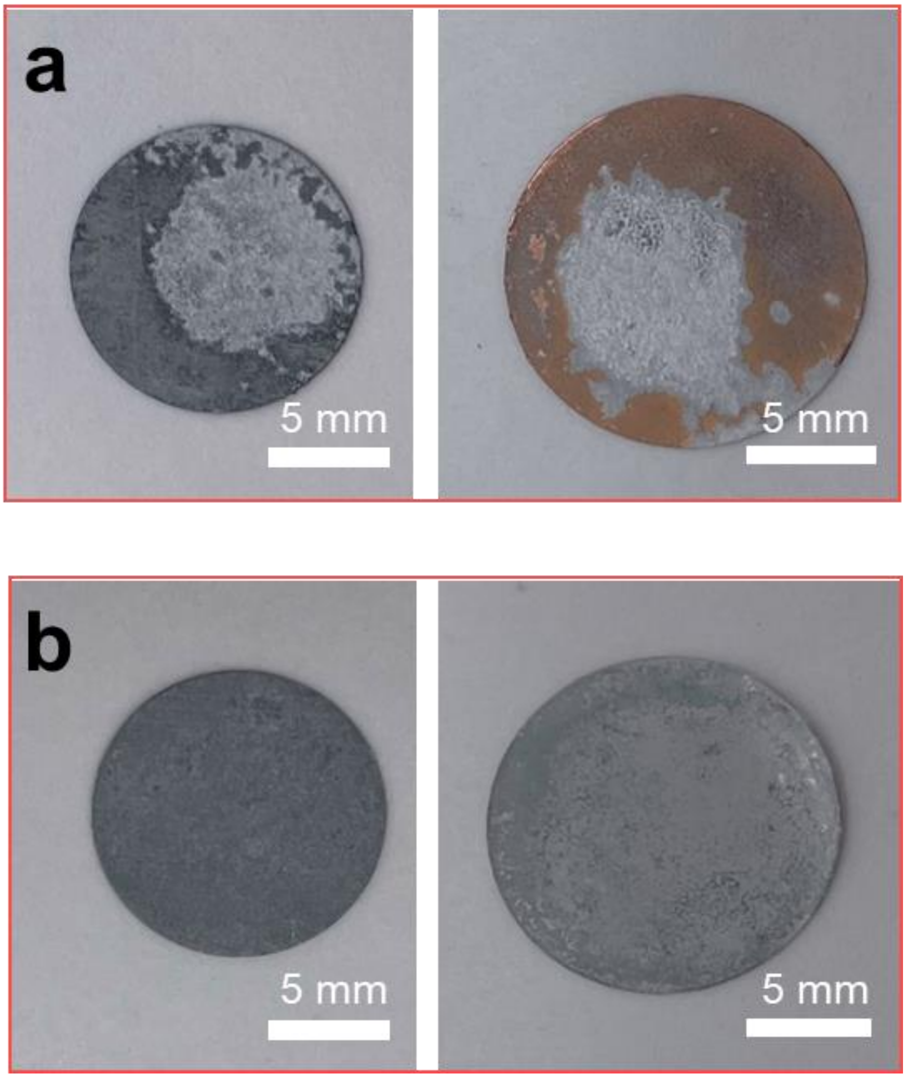

Supplementary Figure S38. Optical images of Zn metal and Cu anode after 100 cycles at 1 mA cm^{-2}, with a capacity of 1 mA h cm^{-2} in (a) the liquid electrolyte and (b) the HA gel electrolyte.

Supplementary Figure S39

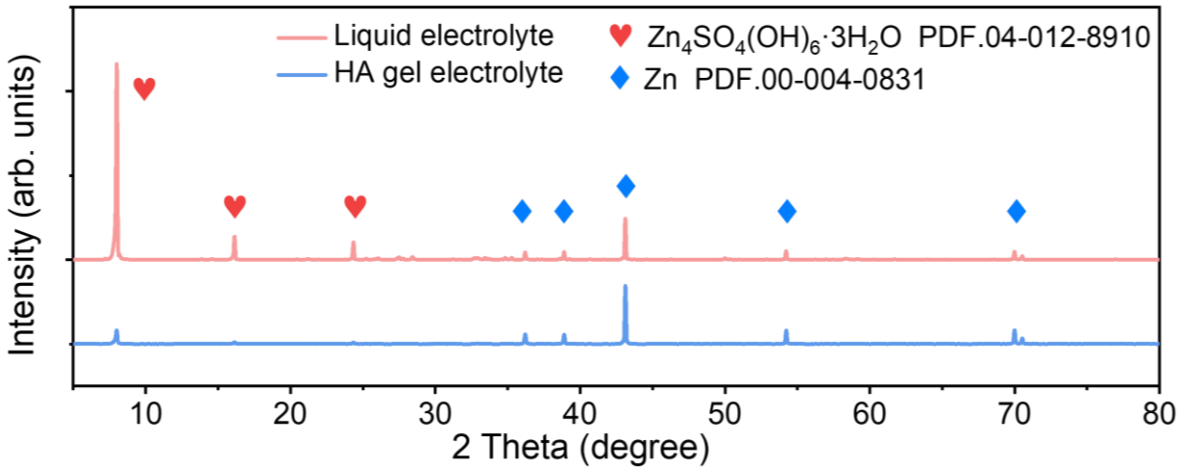

Supplementary Figure S39. XRD spectra of Zn metal after 100 cycles in liquid and HA gel electrolytes at 1 mA cm^{-2} with a capacity of 1 mA h cm^{-2}.

Supplementary Figure S40

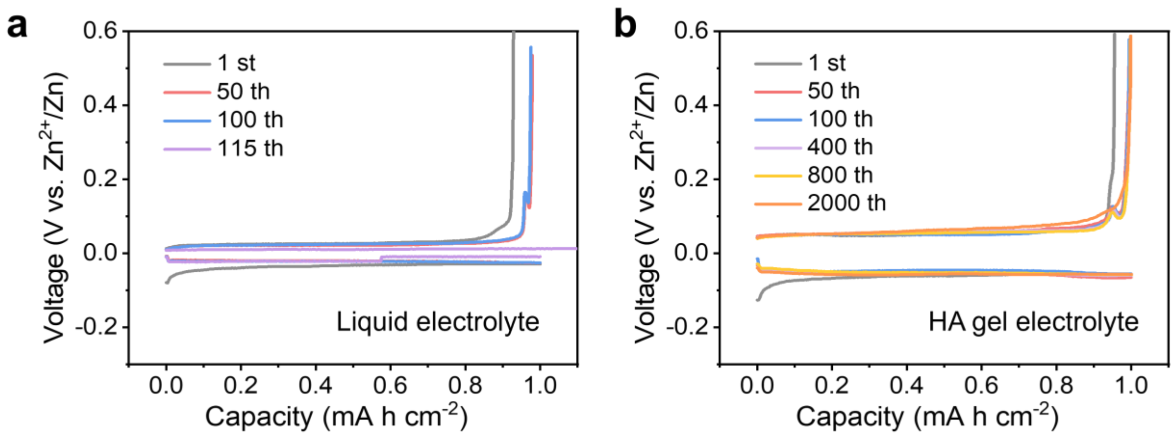

Supplementary Figure S40. Selected capacity-voltage profile of Zn//Cu cells using (a) liquid electrolyte and (b) the HA gel electrolyte at a current density of 1 mA cm^{-2} with a capacity of 1 mA h cm^{-2}.

Supplementary Table S5. The comparison of cumulative capacity and average CEs with previously reported gel electrolytes. Abbreviations of gel electrolytes are as follows: Hyaluronic acid (HA), poly(ethylene glycol) (PEG), poly(acrylamide-co-[2-(methacryloyloxy)ethyl]dimethyl-(3-sulfopropyl)) (PASHE), carboxyl-grafted polyvinyl alcohol and xanthan gum (PSX), poly(3-(1-vinyl-3-imidazolio) propanesulfonate) (PVIPS), poly-2-Acrylamido-2-methylpropanesulfonic/polyacrylamide (PAMPS-PAAM), polyacrylamide-poly (ethylene glycol) diacrylate-carboxymethyl cellulose (PMC), poly 2-acrylamido-2-methyl-1-propane sulfonate zinc (PAMPSZn), iota-carrageenan (IC), polyacrylamide (ZS/GL/AN), sorbitol-modified cellulose (Sor-Cel), polyacrylamide (ZL-PAAM), polyacrylamide (PDZ-H).

**Gel electrolytes**	**Zn/Zn Symmetric cell**			**Zn/Cu Asymmetric cell**		**Ref**
	**Testing conditions**	**life (h)**	**Cumulative capacity (Ah cm^{-2})**	**Testing conditions**	**Average Coulombic efficiency (%)**	
**HA**	5 mA cm^{-2}<br>5 mA h cm^{-2}	1200	3	1 mA cm^{-2}<br>1 mA h cm^{-2}	99.7	This work
**PEG**	5 mA cm^{-2}<br>5 mA h cm^{-2}	450	1.125	5 mA cm^{-2}<br>5 mA h cm^{-2}	98.5	1
**PASHE**	2 mA cm^{-2}<br>2 mA h cm^{-2}	200	0.4	4 mA cm^{-2}<br>1 mA h cm^{-2}	99.4	2
**PSX**	1 mA cm^{-2}<br>1 mA h cm^{-2}	300	0.15	1 mA cm^{-2}<br>1 mA h cm^{-2}	99.1	3
**PVIPS**	7.5 mA cm^{-2}<br>7.5 mA h cm^{-2}	~400	1.5	1 mA cm^{-2}<br>1 mA h cm^{-2}	99.6	4
**PAMPS-PAAM**	2 mA cm^{-2}<br>4 mA h cm^{-2}	360	0.36	5 mA cm^{-2}<br>2.5 mA h cm^{-2}	99.1	5
**PMC**	1 mA cm^{-2}<br>1 mA h cm^{-2}	5000	2.5	1 mA cm^{-2}<br>1 mA h cm^{-2}	99.5	6
**PAMPSZn**	1 mA cm^{-2}<br>1 mA h cm^{-2}	~4500	2.25	1 mA cm^{-2}<br>1 mA h cm^{-2}	98.9	7
**IC**	5 mA cm^{-2}<br>5 mA h cm^{-2}	500	1.25	1 mA cm^{-2}<br>1 mA h cm^{-2}	98.5	8
**ZS/GL/AN**	2 mA cm^{-2}<br>2 mA h cm^{-2}	500	0.5	0.5 mA cm^{-2}<br>1 mA h cm^{-2}	99.5	9
**Sor-Cel**	2 mA cm^{-2}<br>1 mA h cm^{-2}	800	0.8	1 mA cm^{-2}<br>1 mA h cm^{-2}	99.6	10
**ZL-PAAm**	1 mA cm^{-2}<br>1 mA h cm^{-2}	400	0.2	0.2 mA cm^{-2}<br>1 mA h cm^{-2}	95	15
**PDZ-H**	5 mA cm^{-2}<br>10 mA h cm^{-2}	800	2	2 mA cm^{-2}<br>4 mA h cm^{-2}	99.5	16

Supplementary Figure S41

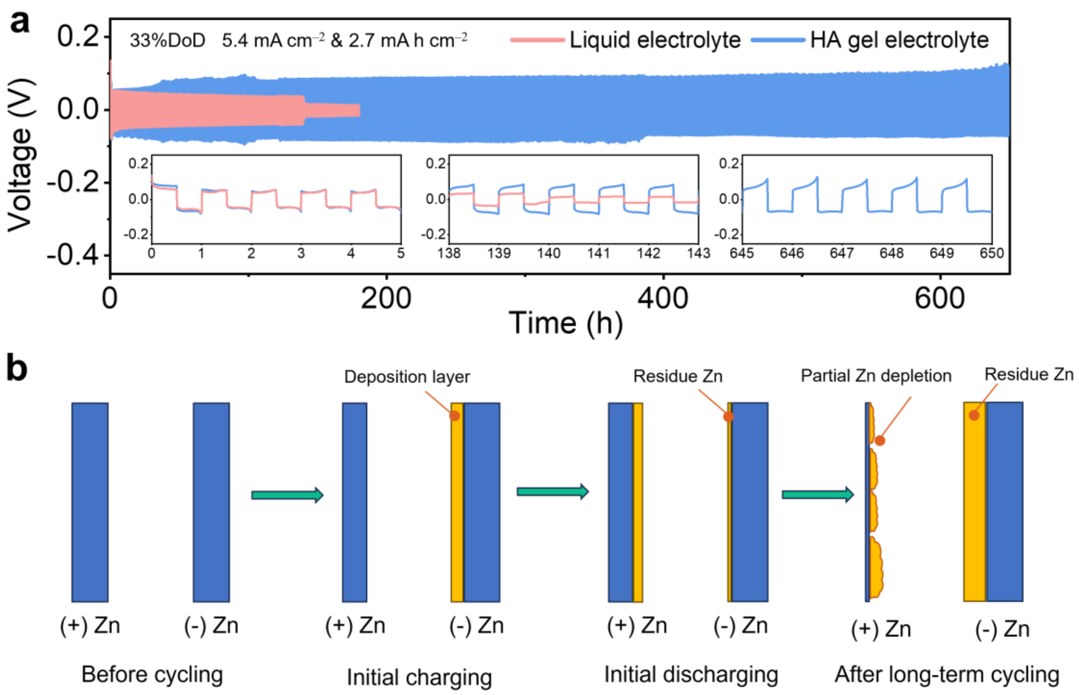

Supplementary Figure S41. Cycle performance of Zn anodes with high Zn utilization rates and its failure mechanism. (a) Cycle performance of Zn//Zn symmetric cells with a relatively high Zn utilisation rate of 33% DoD_{Zn}. (b) Illustration of the change of Zn electrodes during long-term cycling with a high DoD_{Zn}.

The asymmetrical voltage curves at the end of the cycling (Supplementary Figure S41a) of Zn//Zn cells is attributed to the partial depletion of the Zn source of the Zn working electrode. Here, the working electrode is defined as the Zn metal at the positive electrode. In the charging process, Zn is gradually removed from the Zn working electrode. However, during the subsequent discharge process, only a portion of the Zn can retrace the path back to the Zn working electrode. This behavior is due to factors such as unavoidable corrosion reactions and/or irregular Zn deposition, especially at a relatively high DoD of 30% (Supplementary Figure 41b). Noted, though these two problems are significantly suppressed in the HA electrolyte, they cannot be completely eliminated. As a result, over extended cycles, the gradual loss of Zn accumulatively leads to the localised depletion of Zn within the system. This depletion has a detrimental impact on the stability of the electric network and reduces the accessibility of active sites. As a result, the energy barrier for the process of Zn stripping becomes more challenging to overcome, leading to the observed increase of the electrode potential during the charging process.

Supplementary Table S6. Comparison of lifespan of Zn//Zn symmetric cells under high DoD conditions with previously reported gel electrolytes. Abbreviations of gel electrolytes are as follows: Hyaluronic acid (HA), poly(ethylene glycol) (PEG), poly(acrylamide-co-[2-(methacryloyloxy)ethyl]dimethyl-(3-sulfopropyl)) (PASHE), carboxyl-grafted polyvinyl alcohol and xanthan gum (PSX), poly(3-(1-vinyl-3-imidazolio) propanesulfonate) (PVIPS), polyacrylamide (PAM-in situ).

**Gel electrolytes**	**DOD (%)**	**Testing conditions**	**Thickness of Zn anode (um)**	**Life (h)**	**Cycle number**	**Ref**
**HA**	80	6.5 mA cm^{-2} 6.5 mA h cm^{-2}	16	250	125	This work
**HA**	33	5.4 mA cm^{-2} 2.7 mA h cm^{-2}	16	650	650	This work
**PEG**	62.5	10 mA cm^{-2} 10 mA h cm^{-2}	powder	100	50	1
**PASHE**	80.9	20 mA cm^{-2} 10 mA h cm^{-2}	20	100	100	2
**PSX**	34	1 mA cm^{-2} 2 mA h cm^{-2}	10	80	20	3
**PVIPS**	48	7.5 mA cm^{-2} 7.5 mA h cm^{-2}	27	400	200	4
**PAM-in situ**	87	40 mA cm^{-2} 40 mA h cm^{-2}	80	240	120	17

Supplementary Figure S42

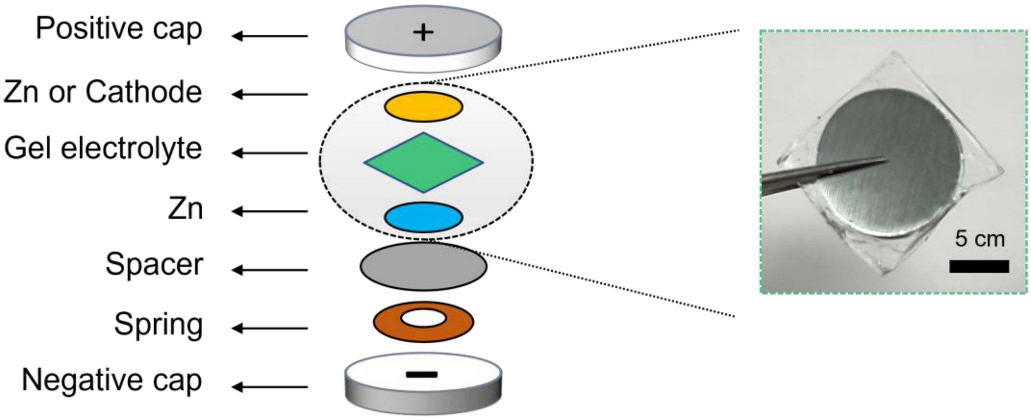

Supplementary Figure S42. Schematic diagram of Zn-based coin cells using the HA gel electrolyte. Optical photo (right) shows the Zn/hydrogel electrolyte/Zn configuration.

Supplementary Figure 43

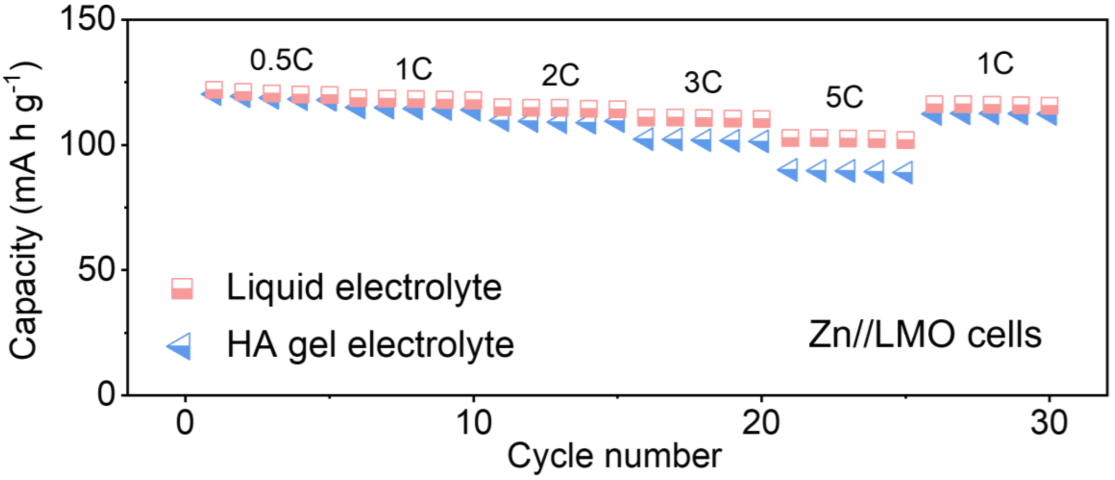

Supplementary Figure S43. Rate capability of Zn//LMO cells in liquid and HA gel electrolytes.

Supplementary Figure S44

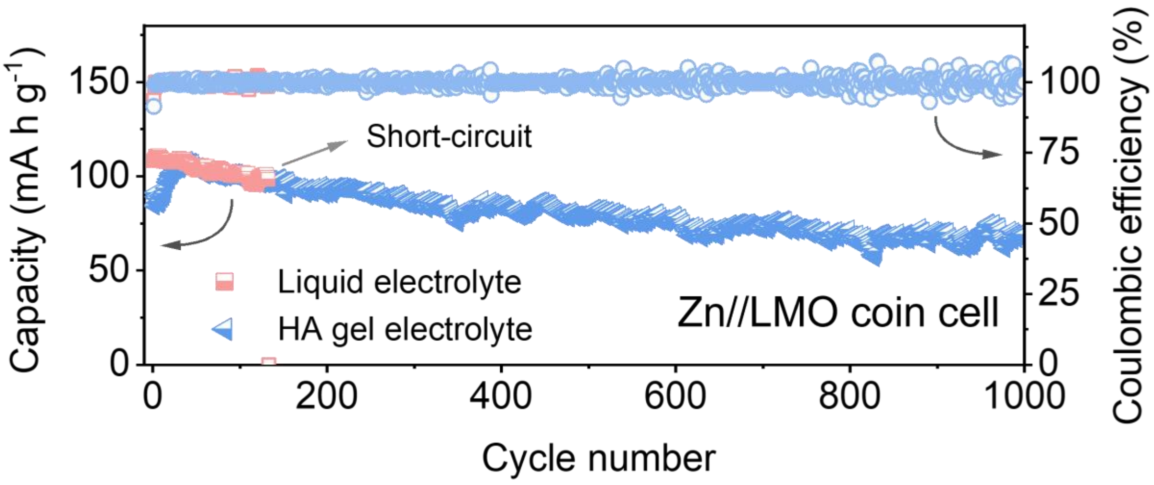

Supplementary Figure S44. Long-term cycle performance of Zn//LMO cells at a current rate of 3C in liquid and HA gel electrolytes.

Supplementary Figure S45

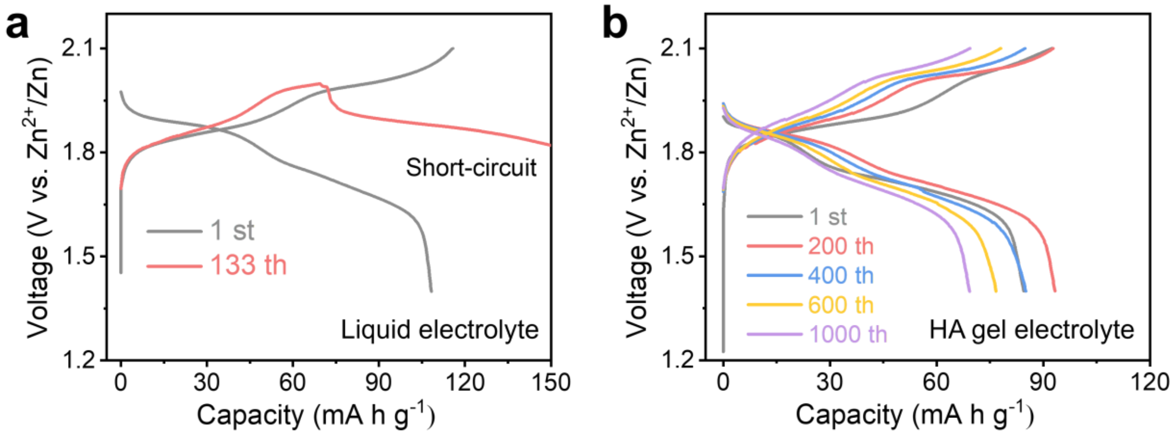

Supplementary Figure S45. Selected capacity-voltage profile of Zn//LMO cells in (a) the liquid electrolyte and (b) the HA gel electrolyte.

Supplementary Figure S46

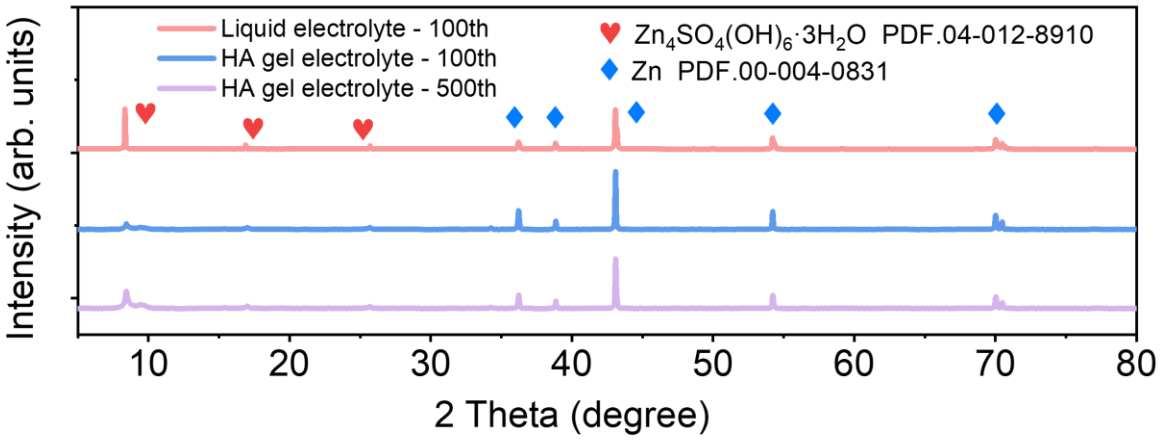

Supplementary Figure S46. XRD spectra of cycled Zn metals in Zn//LMO full cells at 3C using liquid and HA gel electrolytes.

Supplementary Figure S47

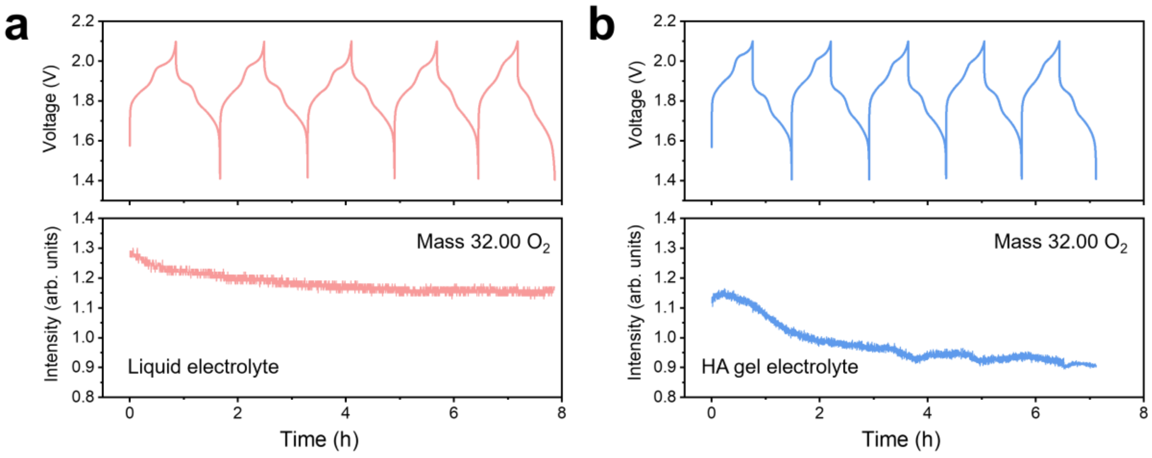

Supplementary Figure S47. Oxygen evolution in Zn//LMO cells. The O_2 spectra obtained from Differential Electrochemical Mass Spectrometry of Zn//LMO cells upon charge/discharge at a current rate of 1C in (a) the liquid electrolyte and (b) the HA gel electrolyte.

Supplementary Figure S48

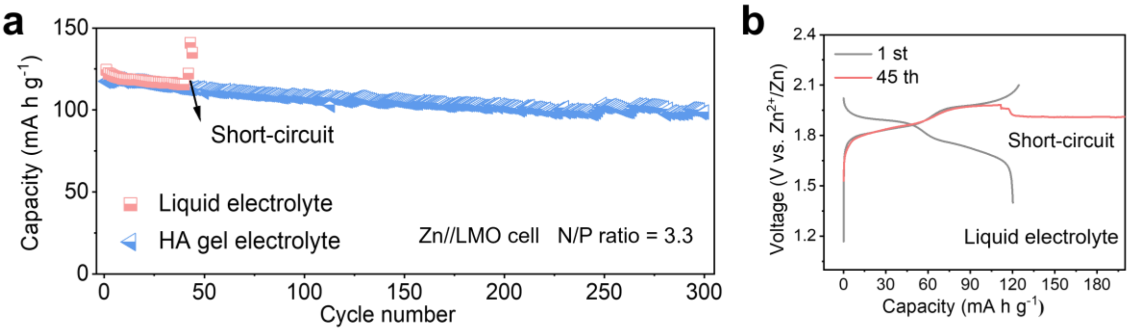

Supplementary Figure S48. Cycle performance of Zn//LMO cells with a low N/P ratio and the failure mechanism in the liquid electrolyte. (a) Long-term cycle performance of Zn//LMO cells with a N/P ratio of 3.3 in liquid and HA gel electrolytes at a current rate of 1C. (b) Selected capacity-voltage profile of Zn//LMO coin cells using the liquid electrolyte.

Supplementary Figure S49

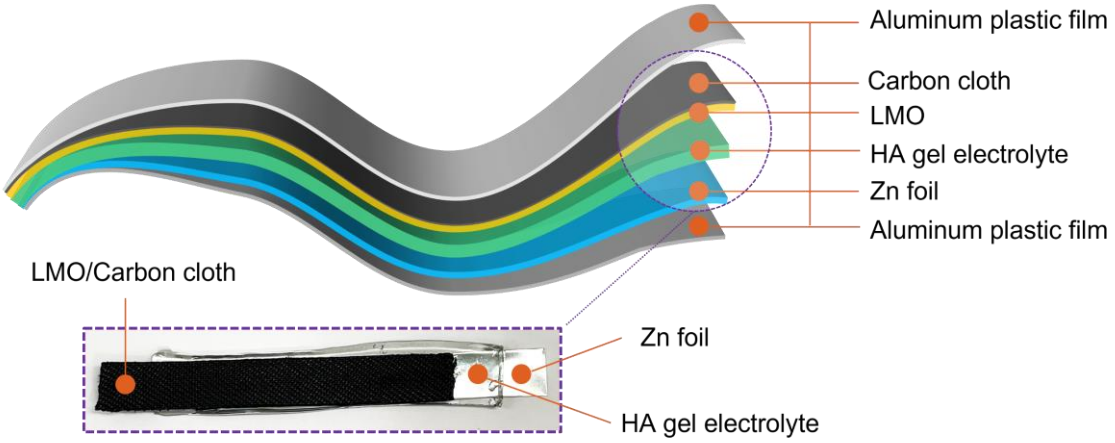

Supplementary Figure S49. Schematic diagram of flexible Zn//LMO battery using the HA gel electrolyte. An optical photo (below) shows the cathode/hydrogel electrolyte/Zn configuration.

Supplementary Figure S50

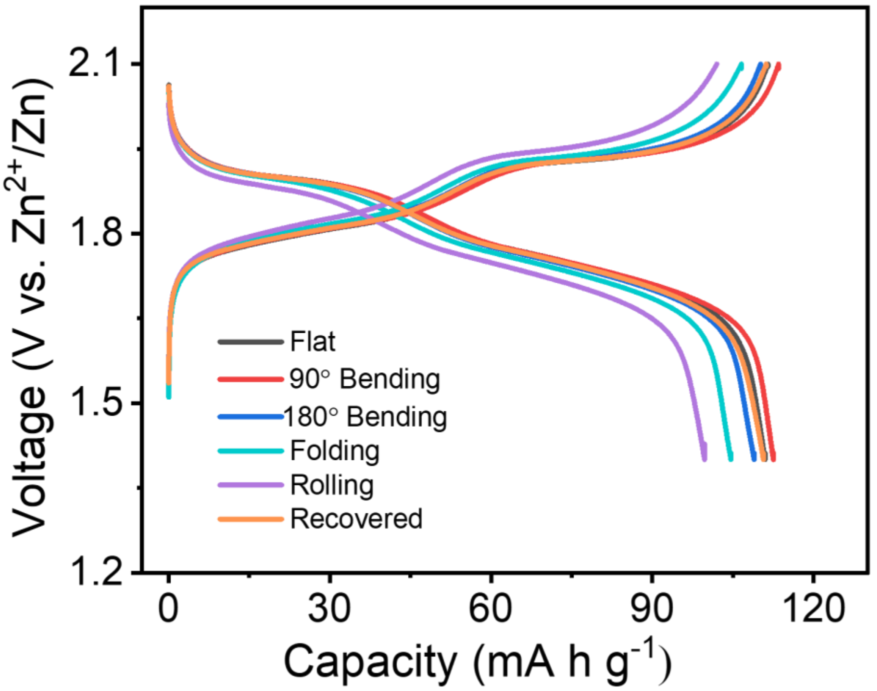

Supplementary Figure S50. Selected capacity-voltage profile of HA gel electrolyte-supported flexible Zn//LMO battery under various mechanical deformation conditions.

Supplementary Figure S51

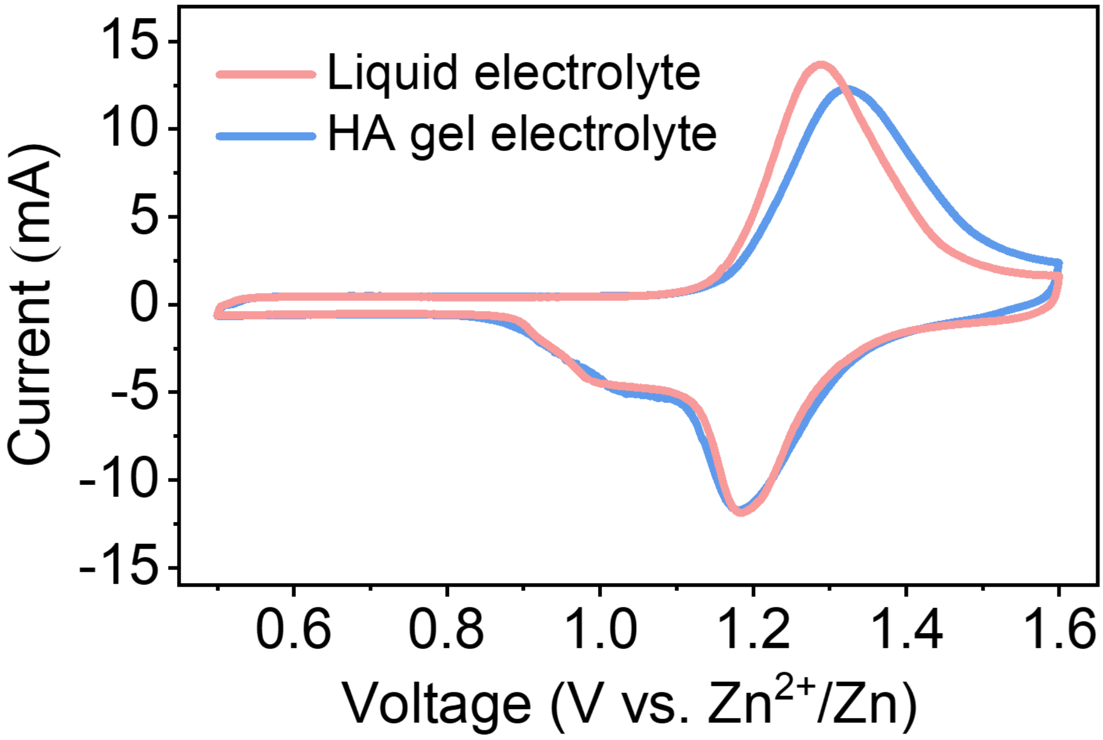

Supplementary Figure S51. Cyclic voltammograms of Zn//I_2 cells in liquid and HA gel electrolytes with a voltage range of 0.5 – 1.6 V at a scanning rate of 1 mV s^{-1}.

Supplementary Figure S52

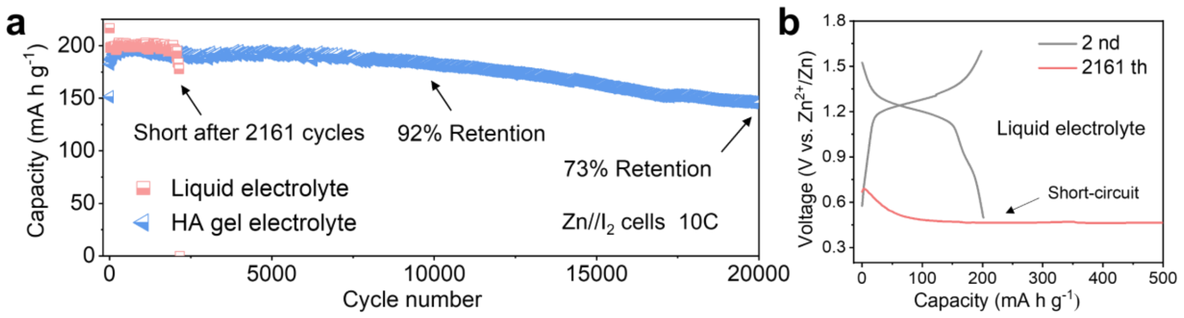

Supplementary Figure S52. Long-term cycle performance of Zn//I_2 cells and the failure mechanism in the liquid electrolyte. (a) Long-term cycle performance of Zn//I_2 cells in liquid and HA gel electrolytes at a current rate of 10C. (b) Selected capacity-voltage profile of Zn//I_2 cells using the liquid electrolyte.

Supplementary Figure S53

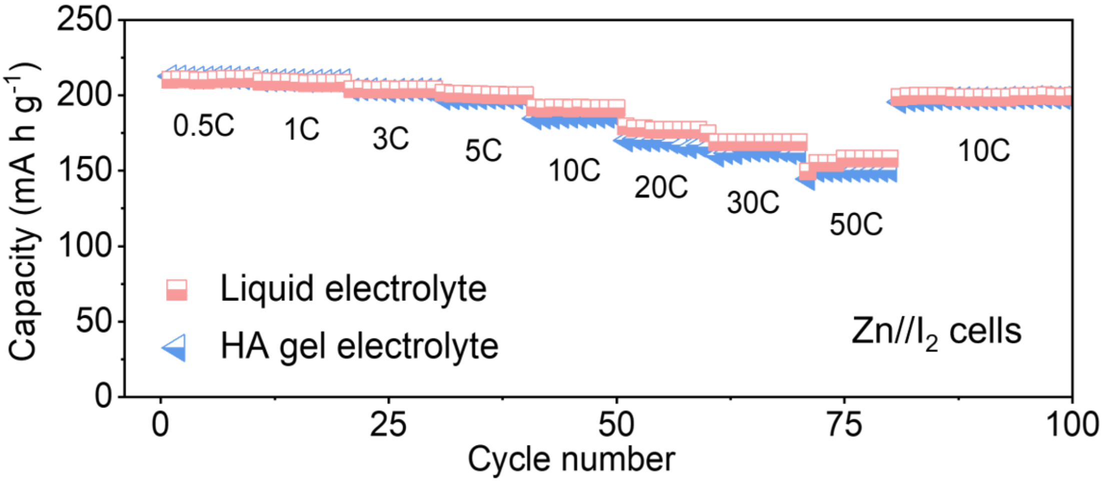

Supplementary Figure S53. Rate capability of $Zn//I_2$ cells in liquid and HA gel electrolytes.

Reference

1 Liu, Q., Yu, Z., Zhou, R. & Zhang, B. A semi-liquid electrode toward stable Zn powder anode. *Adv. Funct. Mater.* **33**, 2210290 (2022).

2 Zhang, W. *et al.* Kinetics-boosted effect enabled by zwitterionic hydrogel electrolyte for highly reversible zinc anode in zinc-ion hybrid micro-supercapacitors. *Adv. Energy Mater.* **12**, 2202219 (2022).

3 Fu, C. *et al.* Modulation of hydrogel electrolyte enabling stable zinc metal anode. *Energy Stor. Mater.* **51**, 588-598 (2022).

4 Hao, Y. *et al.* Gel electrolyte constructing Zn (002) deposition crystal plane toward highly stable zn anode. *Adv. Sci.* **9**, 2104832 (2022).

5 Liu, Y. *et al.* Fundamental study on Zn corrosion and dendrite growth in gel electrolyte towards advanced wearable Zn-ion battery. *Chem. Eng. J.* **446**, 137021 (2022).

6 Lin, P. *et al.* Achieving ultra-long lifespan Zn metal anodes by manipulating desolvation effect and Zn deposition orientation in a multiple cross-linked hydrogel electrolyte. *Energy Stor. Mater.* **49**, 172-180 (2022).

7 Cong, J. *et al.* Ultra-stable and highly reversible aqueous zinc metal anodes with high preferred orientation deposition achieved by a polyanionic hydrogel electrolyte. *Energy Stor. Mater.* **35**, 586-594 (2021).

8 Tian, Y., Chen, S., Ding, S., Chen, Q. & Zhang, J. A highly conductive gel electrolyte with favorable ion transfer channels for long-lived zinc-iodine batteries. *Chem. Sci.* **14**, 331-337 (2023).

9 Wei, T. *et al.* Bonding interaction regulation in hydrogel electrolyte enable dendrite-free aqueous zinc-ion batteries from −20 to 60 °C. *Chem. Eng. J.* **434**, 134646 (2022).

10 Quan, Y. *et al.* Sorbitol-modified cellulose hydrogel electrolyte derived from wheat straws towards high-performance environmentally adaptive flexible zinc-ion batteries. *Chem. Eng. J.* **446**, 137056 (2022).

11 Zhang, T. *et al.* Activating titanium metal with H_2 Plasma for the hydrogen evolution reaction. *ACS Appl. Mater. Interfaces* **13**, 24682-24691, (2021).

12 Heard, D. M. & Lennox, A. J. J. Electrode materials in modern organic electrochemistry. *Angew. Chem. Int. Ed.* **59**, 18866-18884, (2020).

13 Li, Q., Han, L., Luo, Q., Liu, X. & Yi, J. Towards understanding the corrosion behavior of zinc-metal anode in aqueous systems: from fundamentals to strategies. *Batteries Supercaps* **5**, 202100417 (2022).

14 Hickling, A. & Salt, F. W. Studies in hydrogen overvoltage at high current densities: Part I—The influence of electrode material, current density, and time, in aqueous solution. *Trans. Faraday Soc.* **36**, 1226-1235 (1940).

15 Zhu, M. *et al.* Antifreezing hydrogel with high zinc reversibility for flexible and durable aqueous batteries by cooperative hydrated cations. *Adv. Funct. Mater.* **30**, 1907218 (2019).

16 Lu, H. *et al.* Multi-component crosslinked hydrogel electrolyte toward dendrite-free aqueous Zn ion batteries with high temperature adaptability. *Adv. Funct. Mater.* **32**, 2112540 (2022).

17 Qin, Y., Li, H., Han, C., Mo, F. & Wang, X. Chemical welding of the electrode-electrolyte interface by Zn-metal-initiated in situ gelation for ultralong-life Zn-ion batteries. *Adv. Mater.* **34**, 2207118 (2022)
