# Supplementary material for: A biocompatible electrolyte enables highly reversible Zn anode for zinc ion battery
Source: Nat Commun. 2023 Oct 16;14:6526. doi: 10.1038/s41467-023-42333-z (PMC10579325; doi:10.1038/s41467-023-42333-z)
Supplement: Supplementary file 3 — Description of Additional Supplementary Files [file 41467_2023_42333_MOESM3_ESM.pdf]

Description of Additional Supplementary Files

Supplementary Data 1

Initial and final configurations of the liquid electrolyte and HA gel electrolyte systems for molecular dynamics simulations. Optimized computational models of $Zn(H_2O)_6^{2+}$, $Zn(HA)(H_2O)_4^{+}$, and $ZnSO_4(HA)(H_2O)_4^{-}$, obtained from density functional theory calculation.
